# Optimised syntheses and purifications of 3-aryl/heterocyclic dihydrobis- and hydrotris-(pyrazolyl)borate ligands as their alkali salts†

**DOI:** 10.1039/d4ra05723f

**Published:** 2024-09-26

**Authors:** Jarrod R. Thomas, Jonathan T. Mifsud, Scott A. Sulway

**Affiliations:** a School of Chemistry, The University of New South Wales (UNSW) Kensington Sydney 2052 Australia s.sulway@unsw.edu.au

## Abstract

Though poly(pyrazolyl)borate ligands, namely dihydrobis(pyrazolyl)borate (Bp) and hydrotris(pyrazolyl)borate (Tp), have been used in coordination chemistry for decades, their synthesis and purification are of great importance, when targeting high purity and yield of metal complexes. As borohydride substitutions is temperature and pyrazolyl dependent, determining the reaction conditions for each ligand is non-trivial and does not always result in a temperature where only the desired products form. Herein we report the purification of two known Tp^R^ (R = phenyl, 2′-thienyl), as their *tris*-substituted temperatures coincide with their *tetrakis*-temperatures *via* fraction crystallisation from MeCN, and an optimised safe procedure for 3-aryl/heterocyclic Bp^R^ ligands (R = 2′-pyridyl, 2′-furyl, 2′-thineyl). These novel techniques allow for the safe isolation of alkali salts of poly(pyrazolyl)borate ligands in high yields and purities without the use of highly toxic thallium(i) salts.

## Abstract 2

The optimised synthesis and purification of poly(pyrazolyl)borate ligands is detailed for their use in coordination chemistry, to which we implore others to adopt.

## Graphical abstract

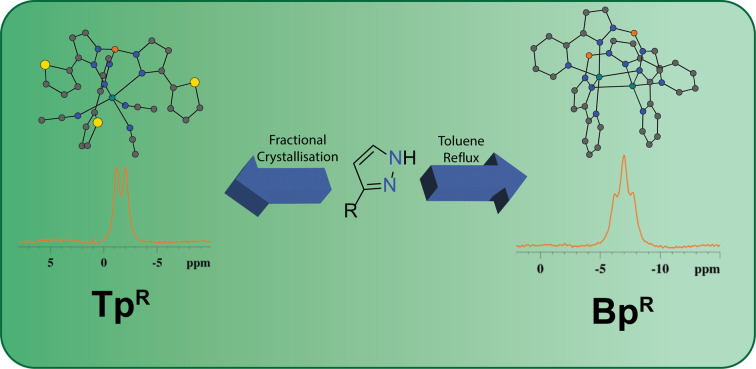

## Introduction

Dihydrobis(pyrazolyl)borate (Bp) and hydrotris(pyrazolyl)borate (Tp, known as scorpionate) ligands were first introduced in the late 1960's by Trofimenko and featured a new synthetic route yielding a B–N bond.^1^ Since their introduction these poly(pyrazolyl)borate ligands have been used extensively throughout coordination chemistry. First generation poly(pyrazolyl)borate featured small alkyl and/or halide substituents on the heterocyclic pyrazolyl groups in the 3rd, 4th and 5th positions.^1*c*^ Coordination of first generation scorpionates to transition metals and lanthanides resulted in the formation of *bis*-capped and *tris*-capped metal complexes, of general form [M(Tp^R^)_2_]^1*b*,*c*^ and [Ln(Tp)_3_],^2^ respectively, regardless of stoichiometry due to high formation constants of the final complexes.^1*b*,*c*^ To synthesis mono-scorpionate metal complexes the use of bulkier substituents were utilized, with first examples of such complexes including [Co(Tp^*t*Bu^)(SCN)] (Tp^*t*Bu^ = hydrotris(3-*tert*-buytlpyrazol-1-yl)borate) and [Co(Tp^Ph^)(SCN)(THF)] (Tp^Ph^ = hydrotris(3-phenylpyrazol-1-yl)borate).^3^ Fine tuning of scorpionate ligands has since become a vast section of coordination chemistry and thus optimising the synthesis of these ligands is of utmost importance. Dihydrobis(pyrazolyl)borate ligands were initially coordinated in a similar matter, forming complexes of the generally form [M(Bp)_2_],^1^ before pyrazole substitution were used to synthesis a range of different metal complexes.^3^

**Scheme 1:**
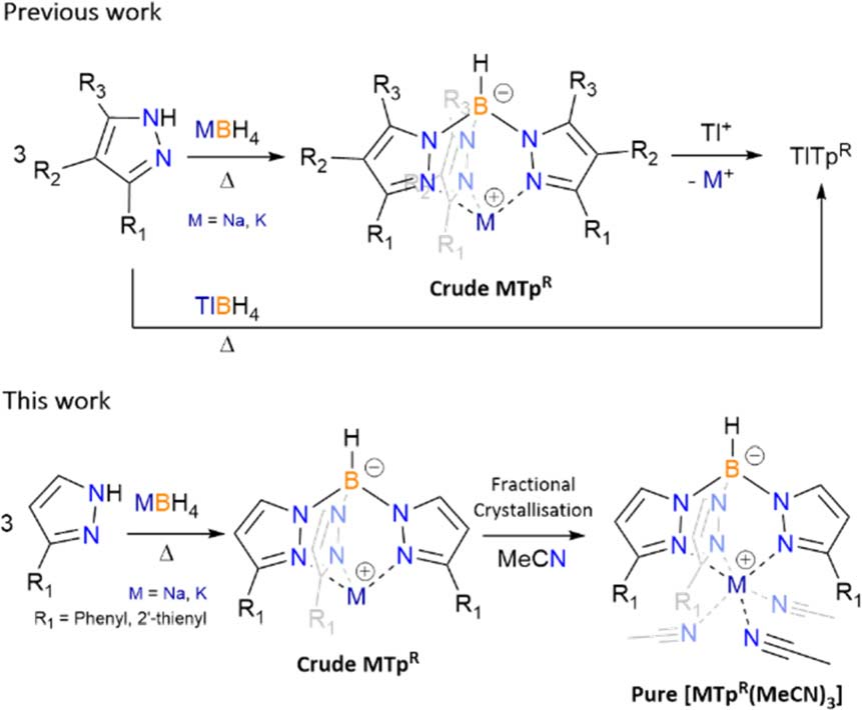
Previous synthesis of scorpionate ligands and new fractional crystallisation of acetonitrile (MeCN) adducts for Tp^Ph^ (R_1_ = phenyl) and Tp^2-Fu^ (R_1_ = 2′-thienyl).

The synthesis of poly(pyrazolyl)borate ligands has remained relatively unchanged, following the Trofimenko procedure which entails heating pyrazolyl reagents in the presence of alkali, or highly toxic thallium(i), borohydrides (Scheme 1).^1,3–6^ Post transmetalation of alkali to thallium(i) salts is another synthetic method used largely for purification of alkali poly(pyrazolyl)borate and the solubility of thallium(i) based scorpionates in organic solvents.^5^ For pyrazolyl substituents with high steric demand some of the syntheses of scorpionate ligands moved to solution-based procedures, using high boiling point solvents, reacting said pyrazole with previously prepared dihydrobis(pyrazolyl)borate.^3^ The two-step processes can seem tedious and for the most part results in low yields of desired products. Recent reporting of the previously inaccessible poly(pyrazolyl)borates has been achieved by *in situ* reaction of sodium pyrazolylides with dichloroborane dimethylsulfide, though isolation of the sodium salts is far from optimised, and instead, transmetalate to the thallium(i) salts for purification.^6^

Due to safety concerns, we restrict the use of thallium(i) salts in our laboratory, thus we targeted alkali salt poly(pyrazolyl)borates as they can be considered a readily safe and useable source of the ligands, and we would also assume the same is true for many other groups. Thus the synthetic routes that allow for the isolation of poly(pyrazolyl)borate ligands, in high purities and moderate yields, is important for further (salt) metathesis reactions. From our previous work, the synthesis of scorpionates that employ 3-R substituted pyrazoles, *e.g.* hydrotris(3-(2′-pyridyl)-pyrazol-1-yl)borate (Tp^2-py^) and hydrotris(3-(2′-furyl)-pyrazol-1-yl)borate (Tp^2-Fu^), as both their sodium and potassium salts can be readily achieved using Trofimenko's procedure (melts at *ca.* 200 °C for 20 hours with a slight excess of pyrazole), resulting in high yields and purity of desired *tris*-products.^7–9^ However, not all pyrazole derivatives are made alike, likely owing to their pK_a_'s, as reagents such as 3-phenylpyrazole and 3-(2′-thienyl)-pyrazole do not have optimal temperatures where *tris*-products exclusively form. From initial syntheses, which are discussed *vide infra* and as reported previously with KTp^Ph^,^7^ once a certain temperature is met the formation of both *tris*- and *tetrakis*-products occurs for the aforementioned pyrazoles. Herein we discuss a new purification technique for scorpionates that encounter oversubstituted contaminates through the isolation of [M(Tp^R^)(MeCN)_3_] (1-Na, M = Na, R = phenyl; 2-Na, M = Na, R = 2′-thienyl (Tp^2-Th^ = hydrotris(3-(2′-thienyl)-pyrazol-1-yl)borate); 2-K, M = K, R = 2′-thienyl), and previous isolation of [K(Tp^Ph^)(MeCN)_3_] (1-K),^7^*via* fractional crystallisation from MeCN.

The same temperature dilemma occurs during the synthesis of dihydrobis(pyrazolyl)borate ligands. Optimal temperatures for the synthesis of Bp ligands using 3-R substituted pyrazolyl reagents employing Trofimenko's method can result in the isolation of crude Bp salts that are contaminated with *tris*-, and in some cases, *tetrakis*-products. The use of these crude products can result in impure products and unwanted side-reactions in subsequent metathetical reactions. Thus, an alternate method for the synthesis of pure MBp^R^ (3-K, M = K, R = 2′-pyridyl (Bp^2-py^ = dihydrobis(3-(2′-pyridyl)-pyrazol-1-yl)borate); 3-Na, M = Na, R = 2′-pyridyl; 3-Li, M = Li, R = 2′-pyridyl; 4, M = K, R = 2′-furyl (Bp^2-Fu^ = dihydrobis(3-(2′-furyl)-pyrazol-1-yl)borate); 5, M = K, R = 2′-thienyl (Bp^2-Th^ = dihydrobis(3-(2′-thienyl)-pyrazol-1-yl)borate)) is illustrated, without the use of purification by thallium(i) abstraction.^6^

## Results and discussion

### Synthesis and spectroscopic characterisation

#### Scorpionate ligands

**Fig. 1:**
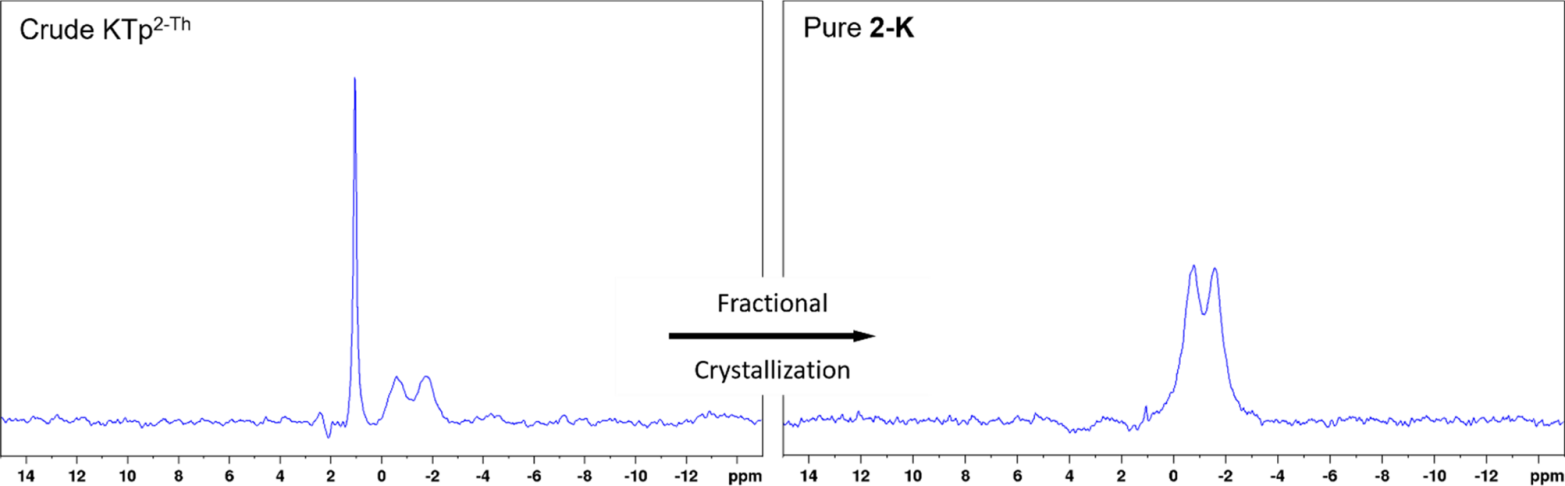
^11^B NMR spectra of the crude and purified product 2-K where pre-purification the presence of *tris*- and tetrakis-products is observed but post fractional crystallisation the desired *tris*-product is solely observed. Baseline corrections have been made to remove undesired borosilicate peak from glassware.

Initial syntheses using Trofimenko's procedure (Scheme 1) for a vast number of scorpionates previously reported, that being Tp, hydrotris(3,5-dimethylpyrazol-1-yl)borate (Tp^Me2^), Tp^2-py^, hydrotris(3-(4′-pyridyl)-pyrazol-1-yl)borate (Tp^4-py^) and Tp^2-Fu^, were all found to have optimal temperatures where *tris*-products form solely with no indication of *tetrakis*-products.^7–9^ These temperatures were found using *in situ* tracking method *via*^11^B NMR spectroscopy.^7^ However when translating Trofimenko's procedure to Tp^2-Th^ and Tp^Ph^, no optimal temperatures could be identified, as stated *vide supra*, with the isolated products from these reaction being mixtures of *tris*- and *tetrakis*-products (ratios are summarised in Table 1). An example of mixed products from melt reactions can be seen in Fig. 1 (left) where once the reaction producing 2-K reaches a temperature of *ca.* 170 °C and is maintained for 20 hours, the ^11^B NMR spectrum shows the Tp^2-Th^ doublet and a downfield singlet due to the presence of *tetrakis*-products. To further optimise these reactions, melt temperatures were raised to 190 °C and reaction times were reduced to 16 hours for all compounds, to limit the presence of bis-substituted products and ensure reagent consumption.

**Table 1:** Ratio of *tris* : *tetrakis* and NMR Data for 1-Na, 1-K, 2-Na and 2-K

Scorpionate	*tris* : *tetrakis*^a^	*δ* (^11^B)/ppm	^1^ *J*_BH_/Hz
1-Na	93 : 7	−1.30	102.9
1-K	67 : 33	−0.91	112.8
2-Na	71 : 29	−1.62	103.9
2-K	83 : 17	−1.90	100.6

a Ratios of *tris* : *tetrakis* is taken pre-fractional crystallisation.

Once crude material is isolate by the parent procedure, NMR spectroscopy can confirm the presence of described products (Fig. 1, ESI Fig. S1–S3†). Many attempts were made to purify the crude material by column chromatography, but no separation was observed between *tris*- and *tetrakis*-products. In trial reactions with crude KTp^2-Th^, it was discovered that 2-K can be fractionally crystallised from MeCN as single crystal X-ray diffraction analysis on these repeated reaction resulted in the repeated isolation of 2-K. Thus for reactions producing NaTp^Ph^, KTp^Ph^, NaTp^2-Th^ and KTp^2-Th^, fractional crystallisations were performed with these crude scorpionates from MeCN yielding crystals of 1-Na, 1-K, 2-Na and 2-K, respectively, over a 2 day period. Yields of the pure MeCN adducts are between 40–60% (from metal borohydride), which are slightly lower than other scorpionates where over substitution is not a problem. Both ^1^H and ^11^B NMR spectra on 1-Na, 1-K, 2-Na and 2-K show only one borate species with the correct multiplicity (Table 1, Fig. 1 and ESI Fig. S4–S7†), and the addition of an MeCN peak at the correct chemical shift and integration. Identification of *ν*_B–H_ and *ν*_C≡N_ is visible in FTIR spectra for the above compounds alongside microanalyses reflecting the correct compositions of *tris*-MeCN adducts.

We feel that this new purification technique is highly important for further transmetalation reaction incorporating these ligands, or any non-trivial scorpionate, to increase yields and purity of metal complexes. It has been stated previously by others that scorpionates can be hard to purify but given their high denticity they will form the desired complexes when crude material is used.^10^ We have had similar purification issues particularly when optimal temperatures cannot be found for purely *tris*-borate formation. Thus the clean and efficient synthesis of 1-M and 2-M present two cases where scorpionate synthesis is not selective but purification is possible.

#### Dihydrobis(pyrazolyl)borate ligands

**Scheme 2:**
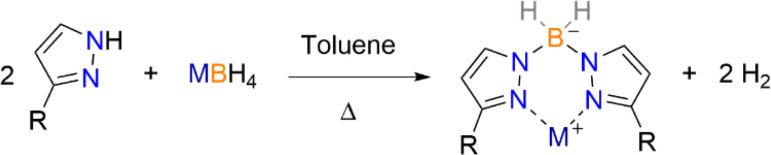
Synthetic route to 3-M (M = Li, Na, K), 4 and 5.

While Trofimenko methods were used initially for the synthesis of first generation Bp ligands, the isolation of purely *bis*-products for 3-aryl/heterocyclic ligands are non-trivial and require temperatures that generally result in the isolation of over substituted products. After repeated attempts of finding optimal temperatures using *in situ* tracking methods,^7^ we opted for solution-based syntheses over Trofimenko's dry melt reactions. Previous reportings' of heteroleptic Bp ligands have been achieved by toluene refluxes.^11^ Analogous procedures for the homoleptic potassium dihydrobis(pyrazolyl)borate ligands 3-K, 4 and 5 were performed with freshly sublimed pyrazole reagents under inert conditions using dry toluene, which after several days precipitates the desired KBp^R^ ligands (Scheme 2). Filtration and appropriate washing result in the isolation of high purity potassium salts of these desired Bp ligands with no evidence of *tris*-products (NMR parameters are characterised is Table 2, see Fig. S10–S12† for NMR spectra). It was discovered that 4 can be recrystallised from pyridine layered with diethyl ether (Et_2_O) and is characterised *vide infra*.

**Table 2:** Yields and NMR parameters for the dihydrobis(pyrazolyl)borate ligands 3-Li, 3-Na, 3-K, 4 and 5

Bis(pyrazolyl)borate	Yield (%)	*δ*(^11^B)/ppm	^1^ *J*_BH_/Hz
3-Li	66	−6.99	—a
3-Na	91	−6.68	92.32
3-K	52	−6.48	91.27
4	50	−6.77	97.38
5	52	−6.94	95.77

a Signal in ^11^B spectra reflects a broad singlet, thus coupling constant could not be accurately determined.

It is worth noting that attempting the same reactions with other alkali borohydrides, namely sodium and lithium, did not yield the same single isolation of preferred *bis*-products for most pyrazole reagents used herein. The single case that resulted in the isolation of *bis*-products for other alkali metals, in moderate yields, was with 3-(2′-pyridyl)-pyrazole yielding LiBp^2-py^ (3-Li) and NaBp^2-py^ (3-Na). There were subtle differences in the workups for said reactions compared to the potassium salts as these Bp salts are soluble in toluene; a reduction of the solvent and washing with Et_2_O to remove unreacted pyrazole reagents yields desired *bis*-products in moderate to high yields (Table 2, ESI Fig. S8 and S9†). Both 3-Li and 3-Na were recrystallized by slow diffusion of Et_2_O from CH_2_Cl_2_, which results in the formation of the dimeric species [M(μ-Bp^2-py^)]_2_ (3-Li_2_, M = Li; 3-Na_2_, M = Na), which have been analysed *vide infra*. These recrystallizations must be performed in anhydrous conditions as the use of ‘wet’ solvents results in the isolation of the water bridged dimers [{M(μ-Bp^2-py^)}_2_(μ-OH_2_)] (see Fig. S29† showcasing the solid-state structure for when M = Na).

### Solid-state characterisation

**Fig. 2:**
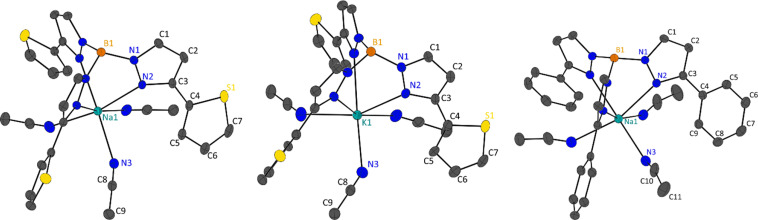
Molecular structure of 2-Na (left), 2-K (middle) and 1-Na (right), shown with 50% ellipsoids, hydrogen atoms have been omitted for clarity. For 2-Na and 2-K molecular structures are grown by (1 + *Y* − *X*, 1 − *X*, +*Z*); (1 − *Y*, +*X* − *Y*, +*Z*) and for 1-Na by (1 − *X*, +*X* − *Y*, +*Z*); (1 + *X* − *Y*, 1 − *X*, +*Z*).

**Fig. 3:**
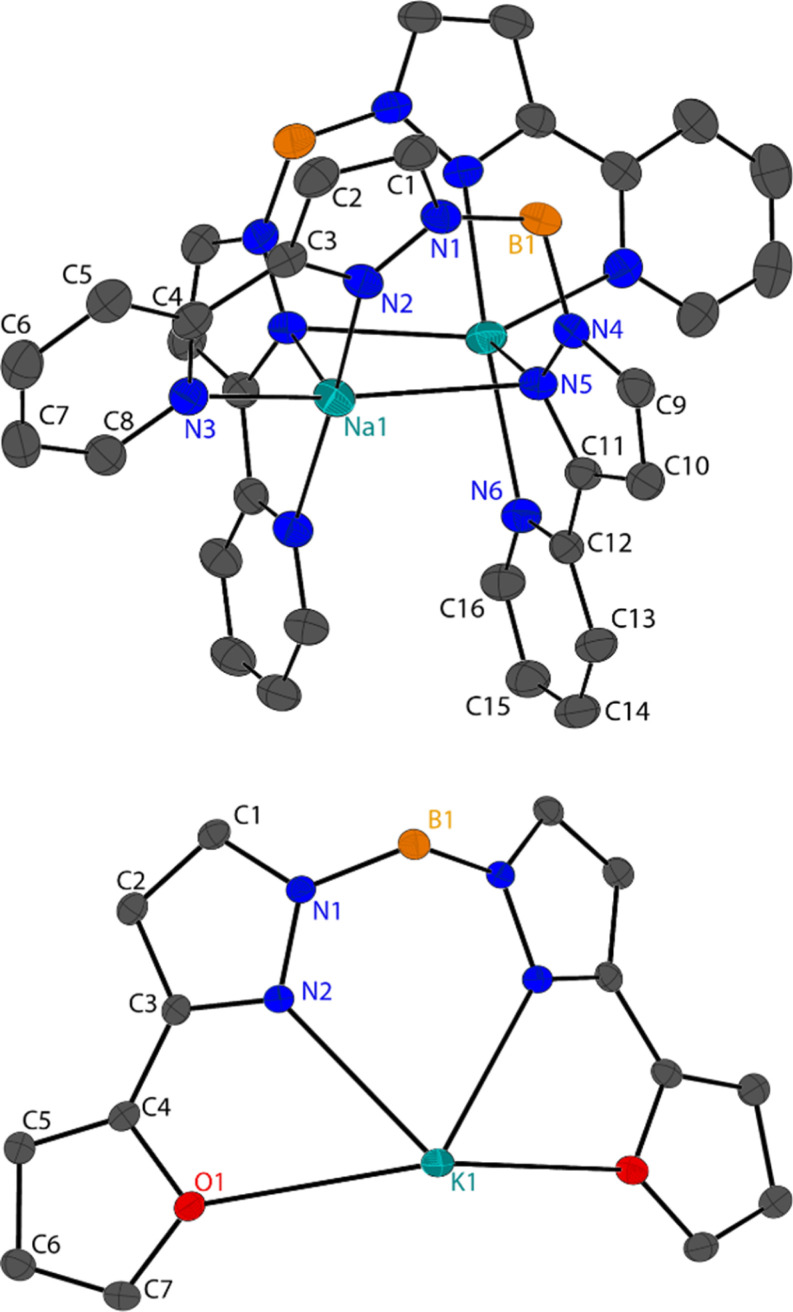
Molecular solid-state structures for 3-M_2_ (top, where M = Na) and 4 (bottom), shown with 50% ellipsoids, hydrogen atoms have been omitted for clarity.

Single crystal X-ray diffraction was performed on the fractionally crystallised 1-Na, 2-Na, 2-K and optimised toluene refluxed products 3-Li_2_, 3-Na_2_ and 4 (X-ray crystallographic data are summarised in Table S1,† molecular structures of these compounds are shown in Fig. 2 and 3 and S25,† asymmetric units are shown within the ESI Fig. S22–S27†), where diffractions studies on 1-K have been previously reported.^7^

#### Scorpionate solid-state structures

Interestingly, all scorpionates pack in trigonal space groups where the highest order rotation axes of *C*_3_ passes through the boron-alkali metal axes (this is also the same direction as the *c*-axis), resulting in the molecular geometry being captured in the crystallographic symmetries. Each tripodal ligand has pyrazolyl nitrogen atoms (N_2_) coordinating to the metal centres, with MeCN ligands coordinated between each pyrazolyl substituent, resulting in pseudo-octahedral (O_h_) coordination environments for all metal complexes. Continuous Shape Measurements (CShM) analysis, employing SHAPE2.1, on the ligand environments show slight deviation from each other, where in general the sodium salts adopt geometries closer to true O_h_ and Tp^2-Th^ are closer to true O_h_ over Tp^Ph^ when comparing analogues alkali metal structures (Table 3, pseudo ligand environments are depicted in Fig. S28†). The distortion in the ligand field environment is attributed to the *fac*-coordination on the scorpionate ligands where a combination of the metal borate (M1⋯B1) distances and conical angles (M1⋯B1⋯N2) result in different coordination distance and angles (Table 2).

**Table 3:** Selected atomic distances, bond lengths, bond angles and torsions angles in 1-M and 2-M (M = Na, K), and CShM values for O_h_ geometry

	1-Na	1-K^a^	2-Na	2-K
M1···B1/Å	3.383(3)	3.738(3)	3.416(5)	3.774(9)
M1–N2/Å	2.5355(13)	2.8325(15)	2.533(2)	2.816(4)
M1–N3/Å	2.5935(14)	2.870(3)	2.535(3)	2.836(4)
M1⋯B1⋯N2/°	48.01(6)	49.2(1)	47.50(8)	48.2(1)
N2–C3–C4–C5/°	163.88(12)	163.88(17)		
N2–C3–C4–S1/°			159.15(19)	161.4(3)
CShM (O_h_)	1.390	2.575	0.644	1.943

a Data taken from literature.^7^

1-Na packs in the trigonal *P*3̄ and isomorphous to our previous reporting for 1-K.^7^ As described, 1/3 of the molecule comprises the asymmetric in 1-Na with one pyrazolyl substituent and one MeCN ligand, and the sodium and boron atoms lying on the *C*_3_ axis, which extends in the *c*-axis direction. Coordination bonds (M1–N2 and M1–N3) are shorter in 1-Na then in 1-K owing to ionic size (Table 3). Within the 1-M structures the scorpionate ligands align down the *c*-direction in columns, all in the same direction with complete overlap, where the adjacent columns are in an antiparallel arrangement (opposing directions). The ‘antiparallel’ arrangement of the columns is likely due to the edge-to-face π-stacking of phenyl rings in adjacent molecules (Fig. S30†) where the distance between the hydrogen atom and the centroid of the phenyl rings is 2.661 Å in 1-Na and 2.832 Å in 1-K.

2-Na and 2-K are also isomorphic, packing in the acentric trigonal space group *R*3*c*. Like 1-M, 2-M structures have 1/3 of the molecular structure in the asymmetric unit with one pyrazolyl substituent and one MeCN ligand, where the *C*_3_ rotational axes pass through the alkali metal and boron atoms. Similarly, due to the ionic radius of the alkali metal used, 2-Na has shorter coordination bonds than 2-K (Table 3). Similar to 1-M, 2-M also form columns of molecules down the *c*-direction, where all columns are ‘parallel’ to each other. Molecules within these columns are not align and instead are generated by glide planes that are parallel with the *C*_3_ axes. Interestingly, the S1 atoms do not coordinate to the metal centres in 2-M, rending the coordination mode of the scorpionate to *κ*^3^, and instead the 2′-thienyl substituent are at torsion angles of 159.15(19)° in 2-Na and 161.4(3)° in 2-K (torsion is taken from N2–C3–C4–S1). In transition metal complexes employing 3-heterocyclic substituted pyrazoles with coordinating atoms, the heterocyclic ring generally have similar torsion angles, however in lanthanide and actinide complexes these functional groups coordinate.^8–10,12,13^ Looking at the supramolecular packing of 2-M, the 2′-thienyl groups are not directed towards anything of chemical significance, however, there is an edge-on π interaction of the heterocyclic ring with MeCN ligands of adjacent molecules. The distances between the terminal carbon of the MeCN ligands and the centroids of the 2′-thienyl substituents are 3.380 Å in 2-Na and 3.328 Å in 2-K (Fig. S31†).

#### Dihydrobis(pyrazolyl)borate solid-state structures

The solid-state structures for 3-Li_2_ and 3-Na_2_ dimers are isostructural but are not isomorphous, however they share the same crystal symmetries and space group of the monoclinic *C*2/*c*. Both structures present the same dimerization, where one Bp^2-py^ ligand and metal centre comprise the asymmetric unit and the Bp^2-py^ ligands bridge the two metal centres (Fig. 3). Both metal centres in 3-M_2_ are five coordinate, however the M1–N5 bond is only included due to the electrostatic nature of these bonds, where the atomic distances are less than 2.9 Å. The M1–N5 bonds are the longest coordination bonds in both structures with the others having ranges of 2.032(4)–2.130(4) and 2.3374(17)–2.5028(18) in 3-Li_2_ and 3-Na_2_, respectively. Assuming the metal centres in 3-M_2_ are 5 coordinate the lowest CShM values present for both structures is for square pyramidal geometry (SP) (Table 4), albeit with very large values (<4.8, ESI Table S3†). If the large M1–N5 bond is not included in the CShM analysis for 3-Li_2_ then the resulting 4 coordinate geometries reflect higher values then for SP (Table S4†), whereas the opposing is true for 3-Na_2_ where the lowest value of 5.368 is seen for Seesaw (*cf.* 5.785 for SP).

**Table 4:** Selected atomic distance, bond lengths, bond angles and torsion angles in 3-M_2_ (M = Li, Na), and CShM values for square pyramidal (SP)

	3-Li_2_	3-Na_2_
M1⋯M1^a^/Å	3.303(8)	3.2724(16)
M1⋯B1/Å	3.676(5)	3.786(3)
M1^a^⋯B1/Å	3.772(5)	3.955(2)
M1–N2/Å	2.032(4)	2.3374(17)
M1–N3/Å	2.130(4)	2.5028(19)
M1–N5/Å	2.869(4)	2.7345(19)
M1^a^–N5/Å	2.053(4)	2.4114(18)
M1^a^–N6/Å	2.121(4)	2.4793(18)
N5–M1–N5^a^	96.86(15)	100.76(6)
M1–N5–M1^a^	82.49(15)	78.70(5)
N2–C3–C4–N3	1.8(3)	0.0(3)
N5–C11–C12–N6	14.8(3)	11.9(3)
CShM (SP)	4.829	5.785

a Molecular geometry grown *via* (1 − *X*, *Y*, ½ − *Z*).

Unlike 1-M and 2-M, 3-M_2_ have the heterocyclic 3-*R*-pyrazolyl substituents coordinating to the metal centres which leads to small torsion angles of said heterocycles with pyrazolyl rings (<15°). The M1–N5 and M1^1^–N5 in 3-M_2_ produce a rectangle that allow for short M1⋯M1^1^ distances of 3.303(8) Å in 3-Li_2_ and 3.2724(16) Å in 3-Na_2_. Two pyridyl groups in 3-M_2_ from different Bp^2-py^ anions within the dimer display π–π interaction, with centroid–centroid distances of 3.465 and 3.935 Å from 3-Li_2_ and 3-Na_2_, respectively (Fig. S32†), with a similar packing seen in adjacent molecules though the centroid distances are much larger (*ca.* 3.5–4.0 Å, Fig. S33†).

Compound 4 packs in the orthorhombic space group *Pnma* and forms a 2-D coordination polymer where one Bp^2-Fu^ anion coordinates to three potassium ions. One half of the KBp^2-Fu^ ion pair in 4 compromises the asymmetric where the boron (including bound hydrogen atoms) and potassium atoms lie on a mirror plane and have half occupancies in the asymmetric unit. The typical coordination bonds from the pyrazolyl substituents are K1–O1 = 3.1369(9) Å and K1–N2 = 2.7997(11) Å. The 2-D nature of the solid-state structures derives from both hydrides bridging to a neighbouring potassium centre which extends the network in the *a*-direction, with a boron–potassium distance of 3.2431(19) Å (Fig. S34†), which is much closer than the K1⋯B1 distance of 3.883(2) Å in the asymmetric unit (see Fig. 3). The network extends in the *c*-direction due to the N1 atoms coordinating to a different adjacent potassium cation, with a coordination distance of 3.3132(11) Å (Fig. S34†).

## Conclusions

The purification of scorpionate ligands that suffer from over substitution at *tris*-substituted activation temperatures, *i.e.* Tp^Ph^ and Tp^2-Th^, by fractional crystallisation has been exemplified through the isolation of 1-Na, 1-K, 2-Na and 2-K of the general formula [M(Tp^R^)(MeCN)_3_]. These *tris*-MeCN adducts have been characterised by FTIR, NMR and X-ray diffraction showing a high purity in bulk materials. Alongside these, the safe and straightforward syntheses of the 3-heteroclylic dihydrobis(pyrazolyl)borate ligands 3-M (M = Li, Na, K) 4 and 5 are exemplified. We implore other groups to use these novel techniques for the purification of other poly(pyrazolyl)borate ligands that suffer from the same over substitution problem, without resorting to the use of highly toxic thallium(i) salts.

## Data availability

The data supporting this article have been included as part of the ESI.†

## Conflicts of interest

There are no conflicts to declare.

## Supplementary Material

RA-014-D4RA05723F-s001

RA-014-D4RA05723F-s002
