# Supplementary material for: Optimised syntheses and purifications of 3-aryl/heterocyclic dihydrobis- and hydrotris-(pyrazolyl)borate ligands as their alkali salts
Source: RSC Adv. 2024 Sep 26;14(42):30713–8. doi: 10.1039/d4ra05723f (PMC11425040; doi:10.1039/d4ra05723f)
Supplement: RA-014-D4RA05723F-s001 [file RA-014-D4RA05723F-s001.pdf]

Supplementary Information (SI) for RSC Advances.
This journal is © The Royal Society of Chemistry 2024

Electronic Supplementary Information (ESI):

Optimised Syntheses and Purifications of 3-Aryl/Heterocyclic dihydro*bis*- and hydro*tris*-(pyrazolyl)borate Ligands as their Alkali Salts

Jarrod R. Thomas [a], *Jonathan T. Mifsud* [a] and *Scott A. Sulway* [a]*

[a] School of Chemistry, The University of New South Wales (UNSW), Kensington, Sydney, 2052, Australia.

* Corresponding author email: s.sulway@unsw.edu.au,

Table of Contents

1. Experimental
2. Spectroscopic Data
3. Single Crystal X-ray Diffraction Collection, Refinement Details and Data
4. SHAPE 2.1 Results
5. References

1. Experimental

General Methods

The starting materials: 2-acetylpyridine, Acetophenone, 2-acetylfuran 2-acetylthiophene, dimethylformamide dimethylacetyl, hydrazine hydrate, metal borohydrides and spectroscopic grade solvents were used as purchased from commercially available sources without further purification. 3-(2′-pyridyl)-pyrazole, 3-phenylpyrazole, 3-(2′-furyl)-pyrazole and 3-(2′-thienyl)-pyrazole were synthesised *via* literature procedures and purified by sublimation.[1] All poly(pyrazolyl)borate reactions were performed using Schlenk techniques under a nitrogen or argon atmosphere. ^{1}H (400 MHz) and ^{11}B (128 MHz) NMR data was collected on a 400 MHz Brucker Advanced III at 298K fitted with BBFO temperature probes. Deuterated solvents were purchased through Sigma-Aldrich, dried over 3 Å sieves and degassed using freeze-pump-thaw methods. ATR-Fourier transform Infrared (ATR-FTIR) spectra were collected on Thermo-Scientific Nicolet iS50 using microcrystalline powders in ambient conditions. Elemental Analysis was carried out at the Mark Wainwright Analytical Centre (MWAC) XRF lab using a Elementar varioMACRO cube (CHN).

Synthesis of [Na(Tp^{Ph})(MeCN)$_3$] (1-Na). Freshly sublimed 3-phenylpyrazole (4.65 g, 32.2 mmol) and $NaBH_4$ (0.393 g, 10.4 mmol) were charged in a flask and placed under an inert atmosphere. Reaction vessel was heated to 190°C and held there for 16 hours. During heating 3-phneylpyrazole became molten and evolution of gas was observed. The reaction was cooled to room temperature producing a solid 'block' of material which was crushed up by sonicating the solids in toluene several times. Crude material was collected *via* filtration and washed with more toluene (2 x 20 mL) and warm hexanes (20 mL). The procedure yielded a white powder that by NMR shows the presences of both Tp^{Ph} and Tkp^{Ph}. Fractional crystallisation of crude powder from MeCN produced colourless, X-ray quality crystals of **1-Na** over a two-day period. Crystals from the first two fractions were isolated by filtration (2.75 g, 4.68 mmol, 45%). Elemental analysis calculated $C_{33}H_{31}B_1N_9Na_1$ (%): C 67.47, H 5.32, N 21.46; found: C 67.04, H 5.07, N 20.96. FTIR (ATR, powder): $\bar{\nu}$ = 2461 (s, $\nu_{B\text{-}H}$), 2256 (s, $\nu_{C\equiv N}$) cm^{-1}. ^{1}H NMR (400 MHz, d_6-acetone, 298 K): δ = 7.78 (d, 3H, $^{3}J_{HH}$ = 2.10 Hz, pzH), 7.75 (dd, 6H, $^{3}J_{HH}$ = 8.32 Hz, $^{4}J_{HH}$ = 1.27 Hz, *o*-PhH), 7.30 (t, 6H, $^{3}J_{HH}$ = 7.55 Hz, *m*-PhH), 7.20 (tt, 3H, $^{3}J_{HH}$ = 7.34 Hz, $^{4}J_{HH}$ = 1.60 Hz, *p*-PhH), 6.47 (d, 3H, $^{3}J_{HH}$ = 2.16 Hz, pzH), 4.90 (q, 1H, $^{1}J_{HB}$ =

97.9 Hz, BH hydride), 2.05 (s, MeCN) ppm; ^{11}B (128 MHz): δ = - 1.30 (d, $^{1}J_{BH}$ = 102.9 Hz, BH borate) ppm.

Synthesis of [K(Tp^{Ph})(MeCN)$_3$] (1-K). Method as described for **1-Na** with freshly sublimed 3-phenylpyrazole (4.20 g, 29.1 mmol) and KBH_4 (0.520 g, 9.64 mmol). Fractional crystallisation from MeCN produced colourless, X-ray quality crystals of **1-K** over a two-day period. Crystals were isolated by filtration (2.84 g, 4.71 mmol, 49%). Elemental analysis calculated $C_{33}H_{31}B_1N_9K_1$ (%): C 65.67, H 5.18, N 20.89; found: C 66.53, H 4.59, N 21.20. FTIR (ATR, powder): $\bar{\nu}$ = 2425 (s, ν_{B-H}), 2255 (s, $\nu_{C\equiv N}$) cm^{-1}. ^{1}H NMR (400 MHz, *d*$_6$-acetone, 298 K): δ = 7.83 (dd, 6H, $^{3}J_{HH}$ = 8.32 Hz, $^{4}J_{HH}$ = 1.26 Hz, *o*-PhH), 7.66 (d, 3H, $^{3}J_{HH}$ = 2.18 Hz, pzH), 7.28 (t, 6H, $^{3}J_{HH}$ = 7.68 Hz, *m*-PhH), 7.14 (tt, 3H, $^{3}J_{HH}$ = 7.34 Hz, $^{4}J_{HH}$ = 1.42 Hz, *p*-PhH), 6.46 (d, 3H, $^{3}J_{HH}$ = 2.17 Hz, pzH), 2.05(s, MeCN) ppm; ^{11}B (128 MHz): δ = -0.91 (d, $^{1}J_{BH}$ = 112.8 Hz, BH borate) ppm.

Synthesis of [Na(*k*^{3}-Tp$^{2\text{-Th}}$)(MeCN)$_3$] (2-Na). Method as described for **1-Na** with freshly sublimed 3-(2'-thienyl)-pyrazole (4.40 g, 29.3 mmol) and $NaBH_4$ (0.358 g, 9.46 mmol). Fractional crystallisation from MeCN produced colourless, X-ray quality crystals of **2-Na** over a two-day period. Crystals were isolated by filtration (2.98 g, 4.92 mmol, 52%). Elemental analysis calculated $C_{27}H_{25}B_1N_9Na_1S_3$ (%): C 53.56, H 4.16, N 20.82, S 15.88; found: C 52.88, H 4.65, N 20.23, S 16.23. FTIR (ATR, powder): $\bar{\nu}$ = 2461 (s, ν_{B-H}), 2258 (s, $\nu_{C\equiv N}$) cm^{-1}. ^{1}H NMR (400 MHz, *d*$_6$-acetone, 298 K): δ = 7.72 (d, 3H, $^{3}J_{HH}$ = 2.12 Hz, pzH), 7.28 (s, 3H, ThH), 7.27 (s, 3H, ThH), 7.00 (t, 3H, $^{3}J_{HH}$ = 4.23 Hz, ThH), 6.36 (d, 3H, $^{3}J_{HH}$ = 2.12 Hz, pzH), 4.80 (q, 1H, $^{1}J_{HB}$ = 98.98 Hz, BH hydride), 2.05 (s, MeCN) ppm;^{11}B (128 MHz): δ = - 1.62 (d, $^{1}J_{BH}$ = 103.9 Hz, BH borate) ppm.

Synthesis of [K(*k*^{3}-Tp$^{2\text{-Th}}$)(MeCN)$_3$] (2-K). Method as described for **1-Na** with freshly sublimed 3-(2'-thienyl)-pyrazole (3.91 g, 26.0 mmol) and KBH_4 (0.452 g, 8.38 mmol). Fractional crystallisation from MeCN produced colourless, X-ray quality crystals of **2-K** over a two-day period. Crystals were isolated by filtration (3.02 g, 4.86 mmol, 58%). Elemental analysis calculated $C_{27}H_{25}B_1N_9K_1S_3$ (%): C 52.17, H 4.05, N 20.28, S 15.47; found: C 52.27, H 3.60, N 19.77, S 15.22. FTIR (ATR, powder): $\bar{\nu}$ = 2443 (s, ν_{B-H}), 2256 (s, $\nu_{C\equiv N}$) cm^{-1}. ^{1}H NMR (400 MHz, *d*$_6$-acetone, 298 K): δ = 7.64 (d, 3H, $^{3}J_{HH}$ = 2.16 Hz, pzH), 7.26 (dd, 3H, $^{3}J_{HH}$ = 3.52 Hz, $^{4}J_{HH}$ = 1.16 Hz, ThH), 7.21 (dd, 3H, $^{3}J_{HH}$ = 5.08 Hz, $^{4}J_{HH}$ = 1.16 Hz, ThH), 6.99 (dd, 3H, $^{3}J_{HH}$ =

5.10 Hz and 3.54 Hz, ThH), 6.37 (d, 3H, $^{3}J_{HH}$ = 2.16 Hz, pzH), 2.06 (s, MeCN) ppm; ^{11}B NMR (128 MHz): δ = -1.29 (d, $^{1}J_{BH}$ = 100.6 Hz, BH borate) ppm.

Synthesis of LiBp$^{2\text{-py}}$ (3-Li). Freshly sublimed 3-(2'-pyridyl)-pyrazole (3.19 g, 22.0 mmol) and $LiBH_4$ (0.239 g, 11.0 mmol) were charged in a flask under an inert atmosphere, toluene (45 mL) was added to the reaction before the solution was refluxed for 20 hours with a slow evolution of gas being observed. The toluene was removed *in vacuo* and replaced with Et_2O (40 mL) to form a suspension. Powder was isolation *via* filtration and washed further with Et_2O (2 x 10 mL), dried under vacuum and isolated as a white powder (2.247 g, 7.29 mmol, 66%). **3-Li** can be recrystallised from CH_2Cl_2 layered with Et_2O to form the dimer [Li(μ-Bp$^{2\text{-py}}$)]$_2$ (**(3-Li)$_2$**). Reliable microanalysis could not be obtained despite repeated attempts. FTIR (ATR, powder): $\bar{\nu}$ = 2381 (s, $\nu_{B\text{-}H}$), 2273 (s, $\nu_{B\text{-}H}$) cm^{-1}. ^{1}H NMR (400 MHz, CD_3OD, 298 K): δ = 8.44 (ddd, 2H, $^{3}J_{HH}$ = 4.99 Hz, $^{4}J_{HH}$ = 1.73 Hz, $^{5}J_{HH}$ = 0.91 Hz, pyH), 8.00 (dt, 2H, $^{3}J_{HH}$ = 8.06 Hz, $^{4}J_{HH}$ = 0.99 Hz, pyH), 7.78 (td, 2H, $^{3}J_{HH}$ = 7.77 Hz, $^{4}J_{HH}$ = 1.79 Hz, pyH), 7.59 (d, 2H, $^{3}J_{HH}$ = 2.13 Hz, pzH), 7.22 (ddd, 2H, $^{3}J_{HH}$ = 7.46 Hz and 5.01 Hz, $^{4}J_{HH}$ = 1.17 Hz, pyH), 6.71 (d, 2H, $^{3}J_{HH}$ = 2.12, pzH) ppm; ^{11}B (128 MHz, d_6-acetone): δ = -6.99 (br. s, $\nu_{1/2} \approx$ 280 Hz, BH_2 borate) ppm.

Synthesis of NaBp$^{2\text{-py}}$ (3-Na). Method as described for **3-Li** with freshly sublimed 3-(2'-pyridyl)-pyrazole (10.0 g, 69.2 mmol), $NaBH_4$ (1.31 g, 34.6 mmol) and toluene (150 mL). Et_2O (50 mL) was used to form a suspension. Powder was isolation *via* filtration and washed further with Et_2O (2 x 20 mL), dried under vacuum and isolated as a white powder (10.2 g, 31.5 mmol, 91%). **3-Na** can be recrystallised from CH_2Cl_2 layered with Et_2O to from the dimer [Na(μ-Bp$^{2\text{-py}}$)]$_2$ (**(3-Na)$_2$**). Reliable microanalysis could not be obtained despite repeated attempts. FTIR (ATR, powder): $\bar{\nu}$ = 2377 (s, $\nu_{B\text{-}H}$), 2285 (s, $\nu_{B\text{-}H}$) cm^{-1}. ^{1}H NMR (400 MHz, d_6-acetone, 298 K): δ = 8.59 (ddd, 2H, $^{3}J_{HH}$ = 4.90 Hz, $^{4}J_{HH}$ = 1.69 Hz, $^{5}J_{HH}$ = 1.00 Hz, pyH), 7.76 (td, 2H, $^{3}J_{HH}$ = 7.56 Hz, $^{4}J_{HH}$ = 1.78 Hz, pyH), 7.72 (dt, 2H, $^{3}J_{HH}$ = 7.95 Hz, $^{4}J_{HH}$ = 1.21 Hz, pyH), 7.54 (d, 2H, $^{3}J_{HH}$ = 2.05 Hz, pzH), 7.19 (ddd, 2H, $^{3}J_{HH}$ = 7.11 Hz and 4.95 Hz, $^{4}J_{HH}$ = 1.52 Hz, pyH), 6.57 (d, 2H, $^{3}J_{HH}$ = 2.05, pzH) ppm; ^{11}B (128 MHz): δ = -6.68 (t, $^{1}J_{BH}$ = 92.32 Hz, BH_2 borate) ppm.

Synthesis of KBp$^{2\text{-py}}$ (3-K). Freshly sublimed 3-(2'-pyridyl)-pyrazole (7.33 g, 50.5 mmol) and KBH_4 (1.33 g, 24.7 mmol) were charged in a flask fitted with a condenser, before being placed under an inert atmosphere. Toluene (50 mL) was added to the reaction before

solution was refluxed for 5 days. Evolution of gas was observed but was slow. After 3 days a white powder precipitated out of the solution. After 5 days the precipitated was isolated *via* filtration and washed with toluene (2 x 15 mL) and further with Et_2O (2 x 15 mL), dried under vacuum and isolated as a white powder (4.40 g, 12.9 mmol, 52%). Reliable microanalysis could not be obtained despite repeated attempts. FTIR (ATR, powder): $\bar{\nu}$ = 2360 (s, $\nu_{B\text{-}H}$), 2272 (s, $\nu_{B\text{-}H}$) cm^{-1}. ^1H NMR (400 MHz, d_6-acetone, 298 K): δ = 8.51 (ddd, 2H, $^3J_{HH}$ = 4.88 Hz, $^4J_{HH}$ = 1.78 Hz, $^5J_{HH}$ = 1.02 Hz, pyH), 7.73 (dt, 2H, $^3J_{HH}$ = 7.99 Hz, $^4J_{HH}$ = 1.27 Hz, pyH), 7.69 (td, 2H, $^3J_{HH}$ = 7.54 Hz, $^4J_{HH}$ = 1.81 Hz, pyH), 7.53 (d, 2H, $^3J_{HH}$ = 2.05 Hz, pzH), 7.12 (ddd, 2H, $^3J_{HH}$ = 7.05 Hz and 4.89, $^4J_{HH}$ =1.56 Hz), 6.57 (d, 2H, $^3J_{HH}$ = 2.05 Hz, pzH), 3.75 (q, 2H, $^1J_{HB}$ = 86.00 Hz, BH_2 hydride) ppm; ^{11}B (128 MHz): δ = -6.48 (t, $^1J_{BH}$ = 91.27 Hz, BH_2 borate) ppm.

Synthesis of KBp$^{2\text{-}Fu}$ (4). Method is the same as **3-K** using freshly sublimed 3-(2'-furyl)-pyrazole (5.00 g, 37.3 mmol) and KBH_4 (1.02 g, 18.6 mmol), refluxed in toluene (50 mL). Product was isolated as a white powder (2.97 g, 9.33 mmol, 50%). **4** can be recrystallised from pyridine layered with Et_2O, yielding the non-solvated product KBp$^{2\text{-}Fu}$. Reliable microanalysis could not be obtained despite repeated attempts. FTIR (ATR, powder): $\bar{\nu}$ = 2344 (s, $\nu_{B\text{-}H}$), 2254 (s, $\nu_{B\text{-}H}$) cm^{-1}. ^1H NMR (400 MHz, d_6-acetone, 298 K): δ = 7.46 (d, 2H, $^3J_{HH}$ = 2.04 Hz, pzH), 7.39 (dd, 2H, $^3J_{HH}$ = 1.84 Hz, $^4J_{HH}$ = 0.84 Hz, FuH), 6.50 (dd, 2H, $^3J_{HH}$ = 3.28 Hz, $^4J_{HH}$ = 0.80 Hz, FuH), 6.39 (dd, 2H, $^3J_{HH}$ = 3.28 Hz and 1.84 Hz, FuH), 6.24 (d, 2H, $^3J_{HH}$ = 2.04 Hz, pzH), 3.66 (q, 2H, $^1J_{HB}$ = 85.39 Hz, BH_2 hydride) ppm; ^{11}B (128 MHz): δ = -6.77 (t, $^1J_{BH}$ = 97.38 Hz, BH_2 borate) ppm.

Synthesis of KBp$^{2\text{-}Th}$ (5). Method is the same as **3-K** using freshly sublimed 3-(2'-thienyl)-pyrazole (5.60 g, 37.3 mmol) and KBH_4 (1.02 g, 18.6 mmol), refluxed in toluene (50 mL). Product was isolated as a white powder (3.80 g, 10.9 mmol, 59%). Reliable microanalysis could not be obtained despite repeated attempts. FTIR (ATR, powder): $\bar{\nu}$ = 2378 (s, $\nu_{B\text{-}H}$), 2247 (s, $\nu_{B\text{-}H}$) cm^{-1}. ^1H NMR (400 MHz, d_6-acetone, 298 K): δ = 7.48 (d, 2H, $^3J_{HH}$ = 2.04 Hz, pzH), 7.20 (dd, 2H, $^3J_{HH}$ = 3.54 Hz, $^4J_{HH}$ = 1.14 Hz, ThH), 7.17 (dd, 2H, $^3J_{HH}$ = 5.12 Hz, $^4J_{HH}$ = 1.12 Hz, ThH), 6.96 (dd, 2H, $^3J_{HH}$ = 5.12 Hz and 3.52 Hz, ThH), 6.27 (d, 2H, $^3J_{HH}$ = 2.04 Hz, pzH), 3.66 (q, 2H, $^1J_{HB}$ = 86.90 Hz, BH_2 hydride) ppm; ^{11}B (128 MHz): δ = -6.94 (t, $^1J_{BH}$ = 95.77 Hz, BH_2 borate) ppm.

2. NMR Spectroscopic Data

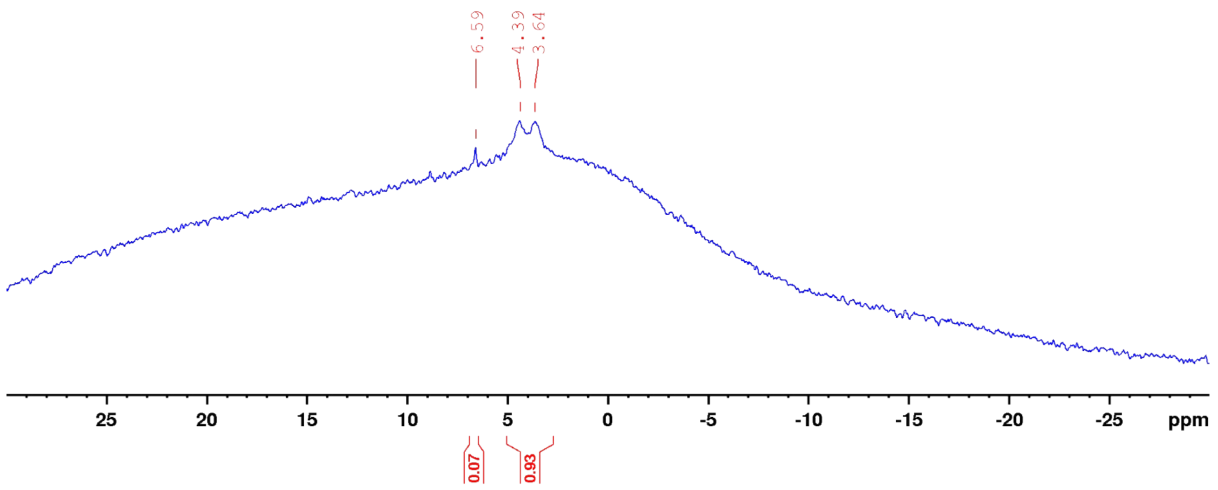

Figure S1. ^{11}B spectra of crude **1-Na**, showing the relative integration of *tetrakis*- (singlet) and tris-products (doublet).

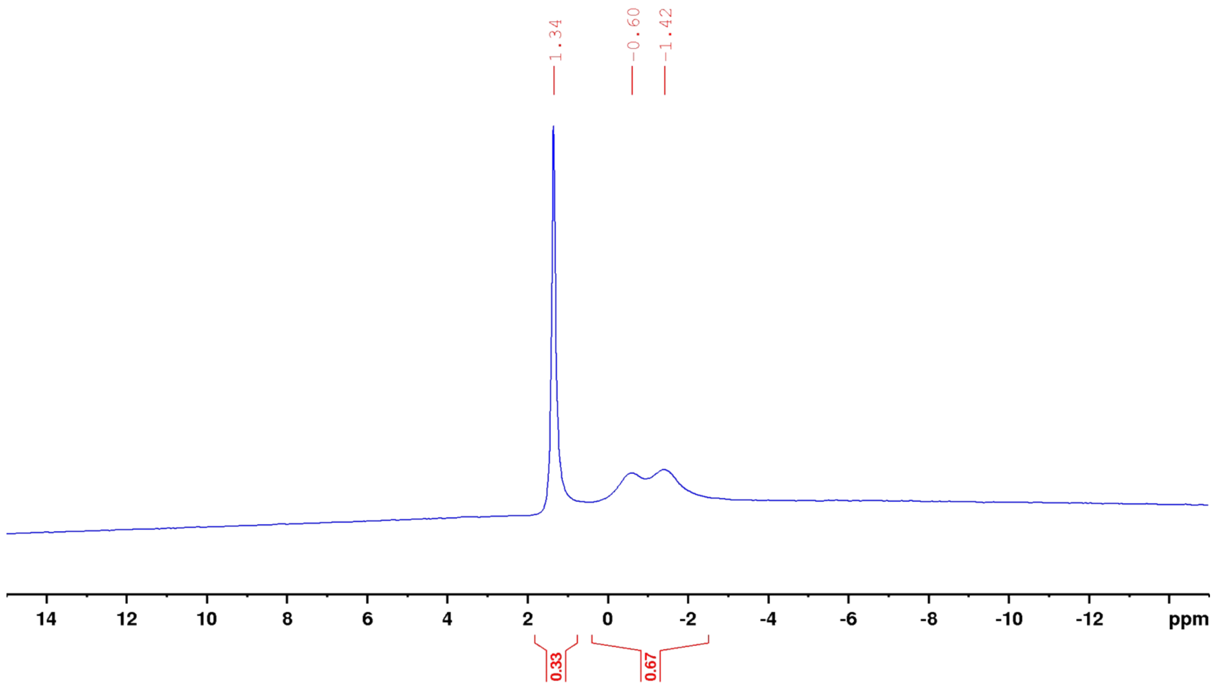

Figure S2. ^{11}B spectra of crude **1-K**, showing the relative integration of *tetrakis*- (singlet) and tris-products (doublet).

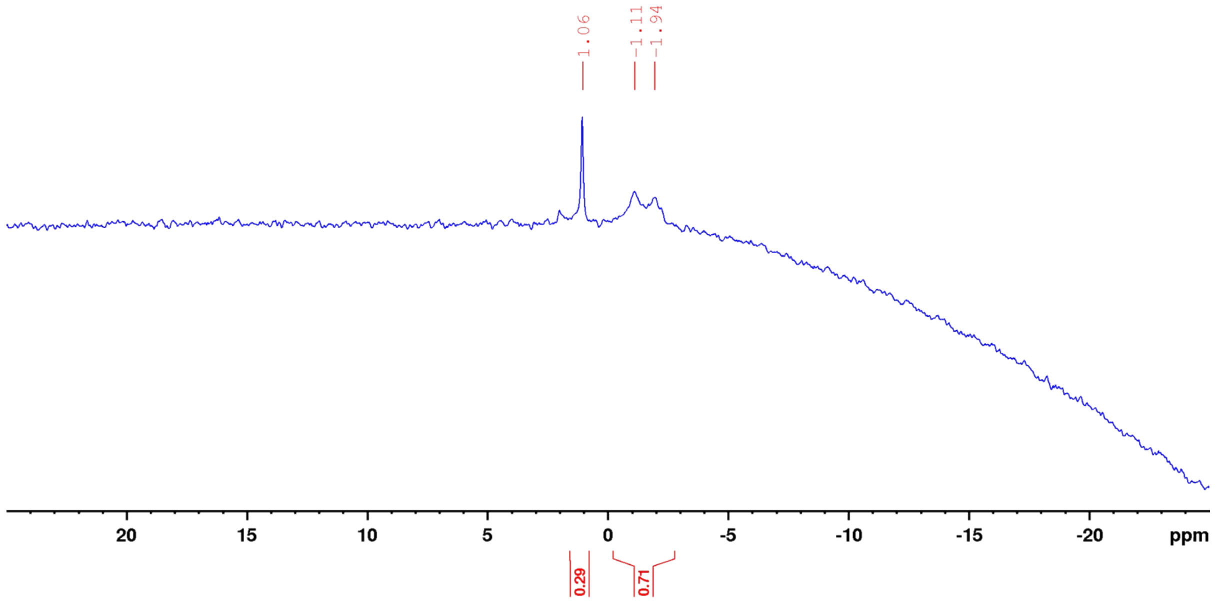

Figure S3. ^{11}B spectra of crude **2-Na**, showing the relative integration of *tetrakis*- (singlet) and *tris*-products (doublet).

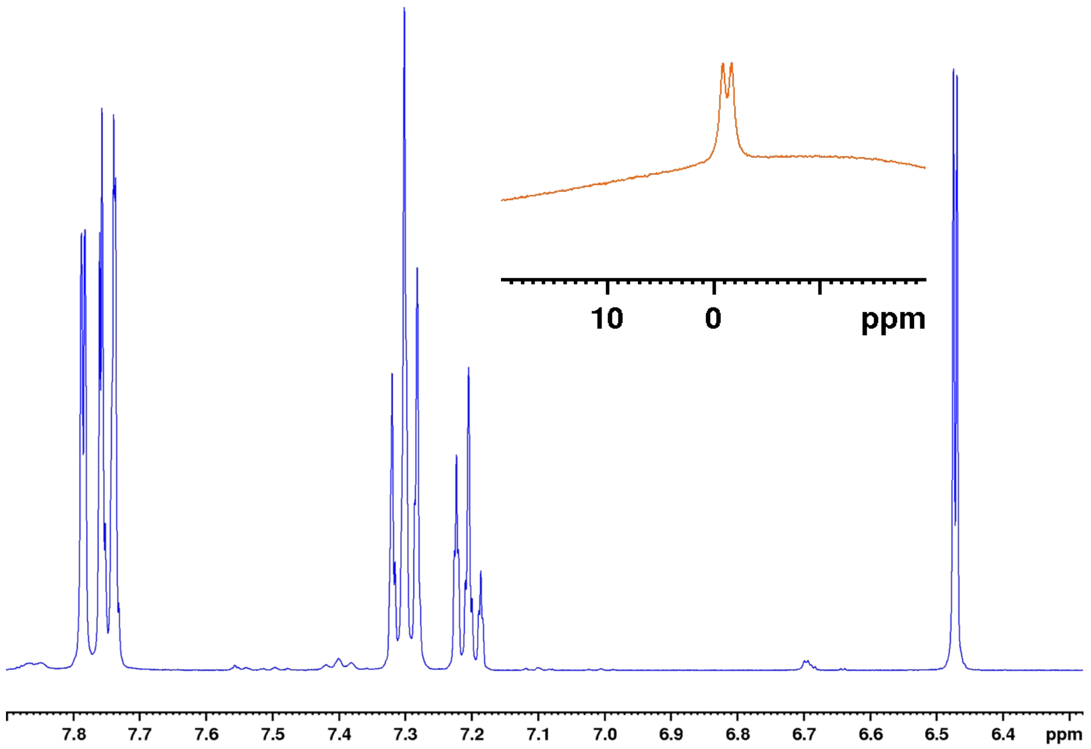

Figure S4. ^{1}H spectrum of fractionally crystalised **1-Na** (blue), with ^{11}B spectrum insert displaying only doublet peak from **1-Na** (orange).

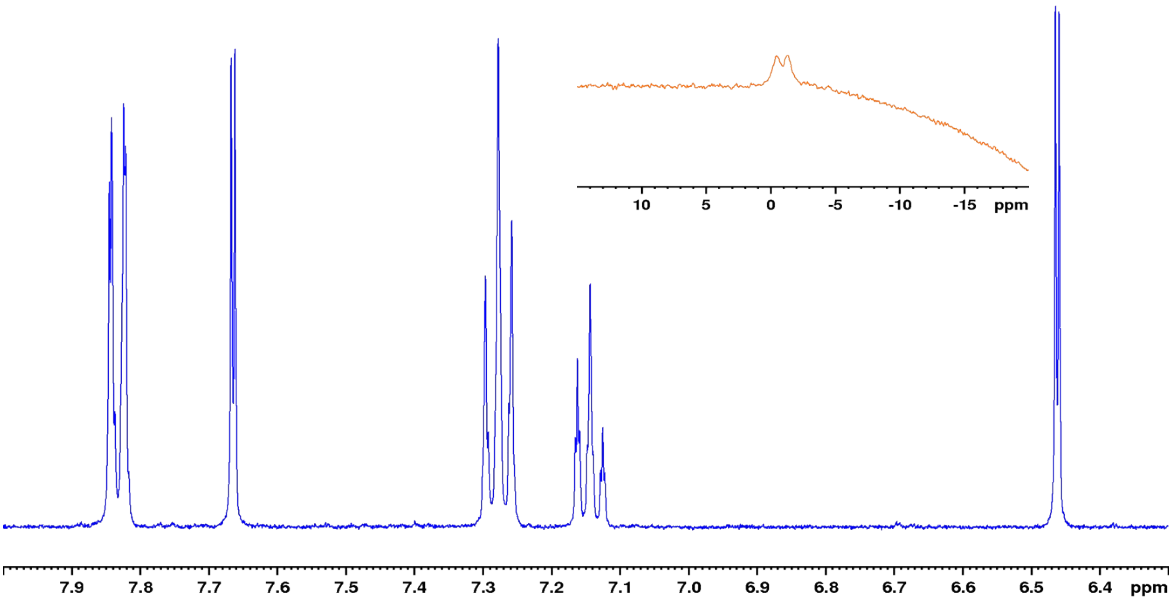

Figure S5. ^{1}H spectrum of fractionally crystalised **1-K** (blue), with ^{11}B spectrum insert displaying only doublet peak from **1-K** (orange).

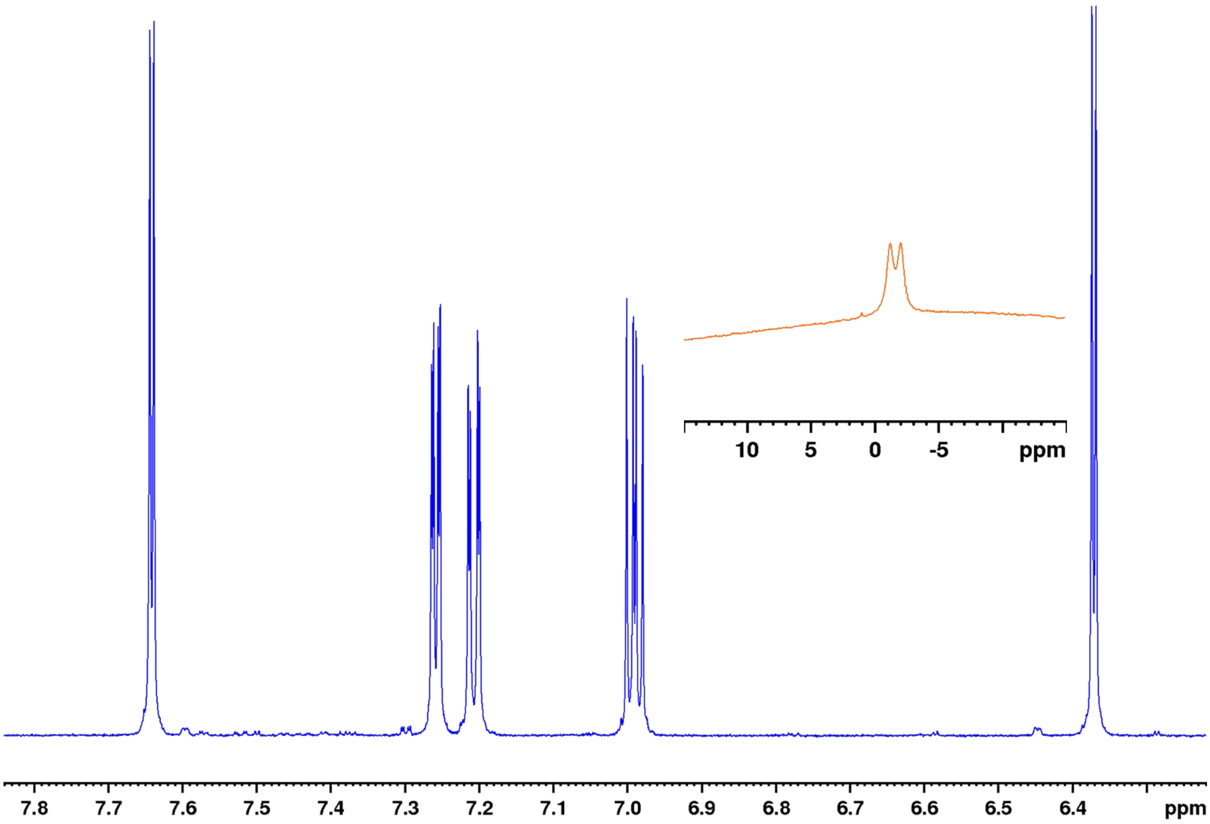

Figure S6. ^{1}H spectrum of fractionally crystalised **2-Na** (blue), with ^{11}B spectrum insert displaying only doublet peak from **2-Na** (orange).

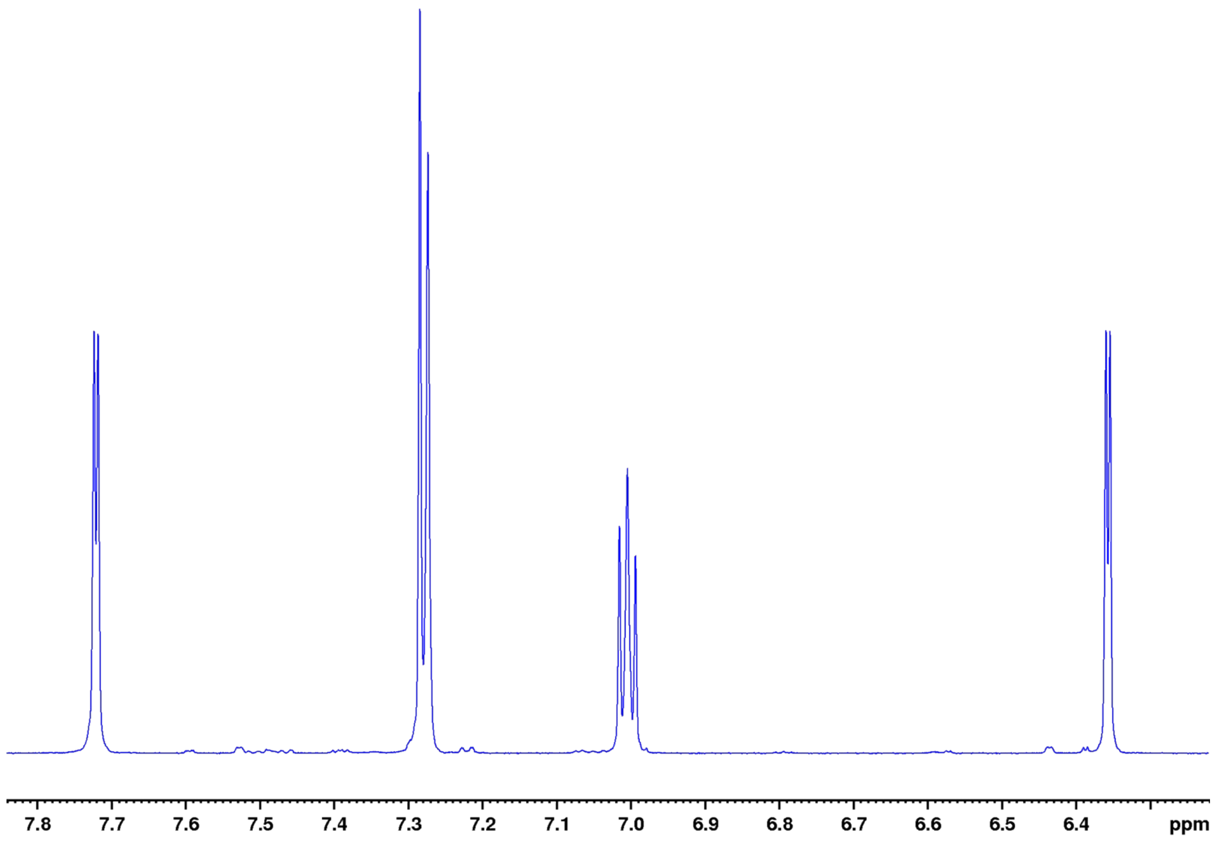

Figure S7. ^{1}H spectrum of fractionally crystalised **2-K** (blue), with ^{11}B spectrum insert displaying only doublet peak from **2-K** (orange).

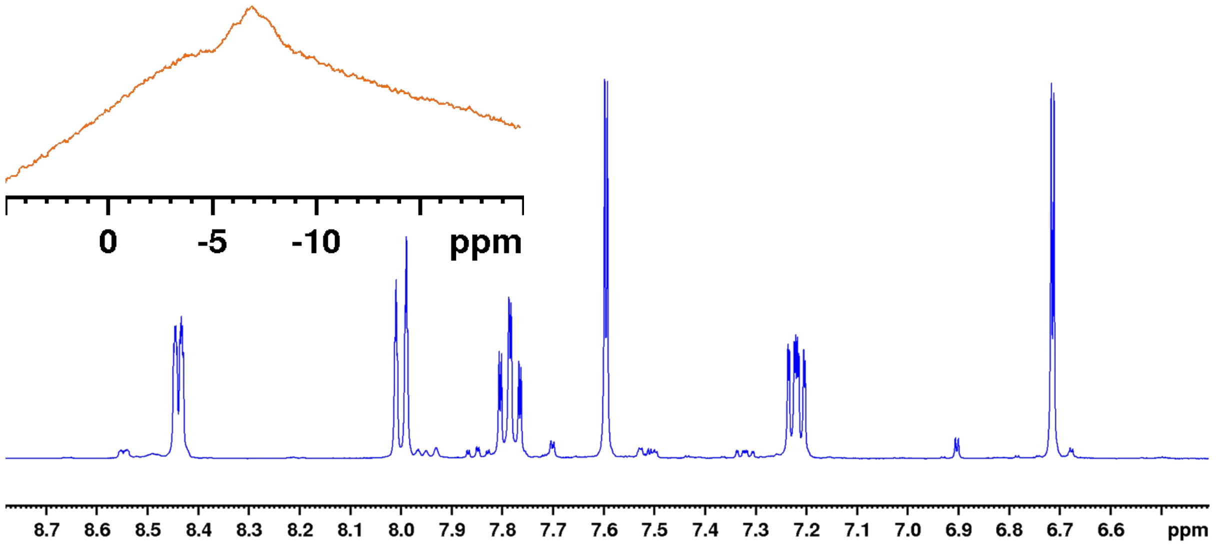

Figure S8. ^{1}H spectrum of **3-Li** (blue), with ^{11}B spectrum insert displaying a broad peak in the correct ppm range for a dihydrobis(pyrazolyl)borate from **3-Li** (orange).

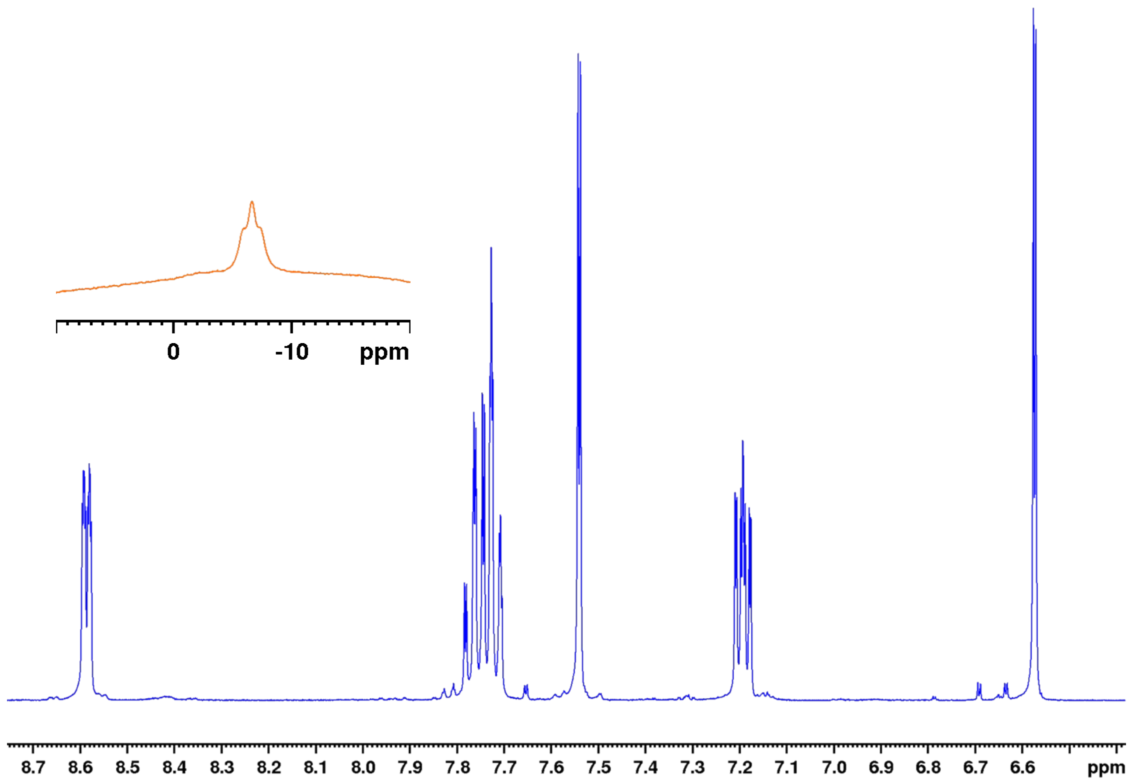

Figure S9. ^{1}H spectrum of **3-Na** (blue), with ^{11}B spectrum insert displaying only triplet peak from **3-Na** (orange).

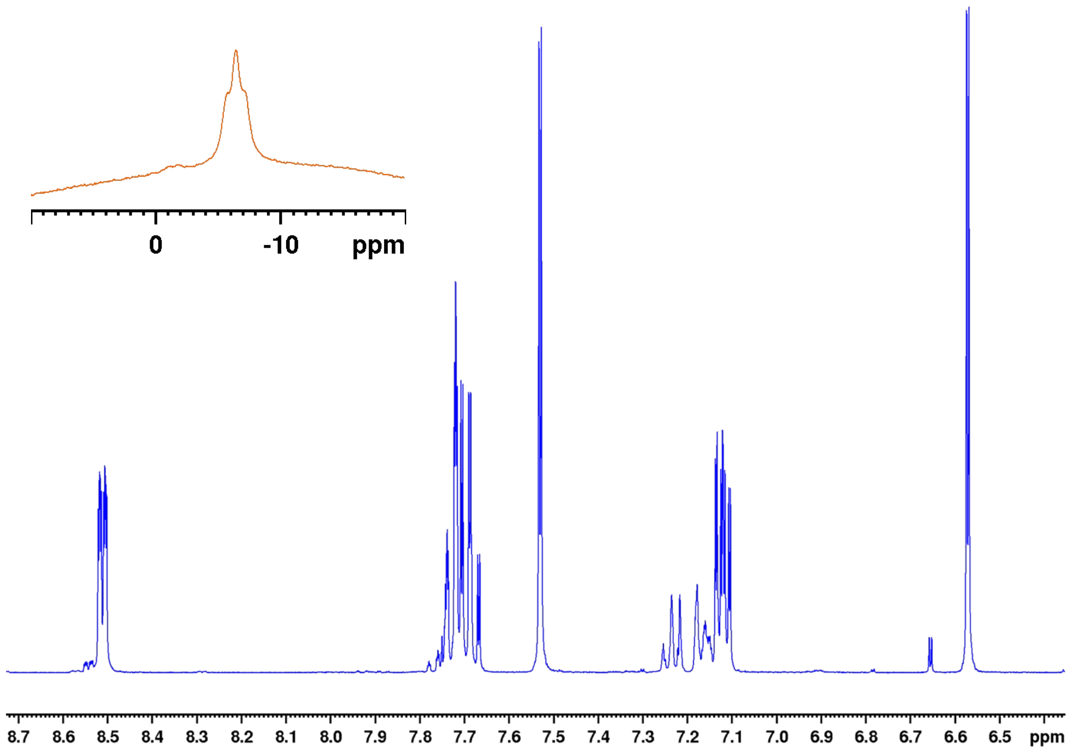

Figure S10 ^{1}H spectrum of **3-K** (blue, toluene impurities are present), with ^{11}B spectrum insert displaying only triplet peak from **3-K** (orange).

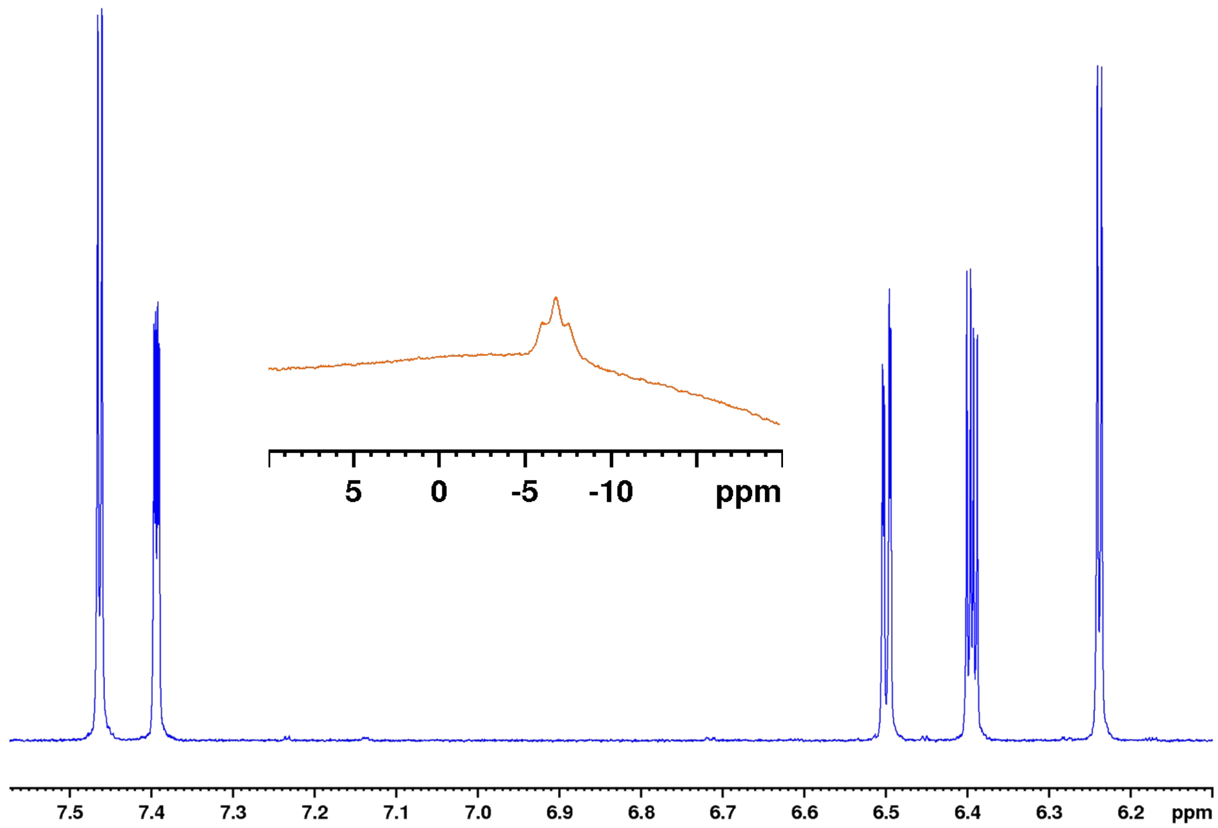

Figure S11. ^{1}H spectrum of **4** (blue), with ^{11}B spectrum insert displaying only triplet peak from **4** (orange).

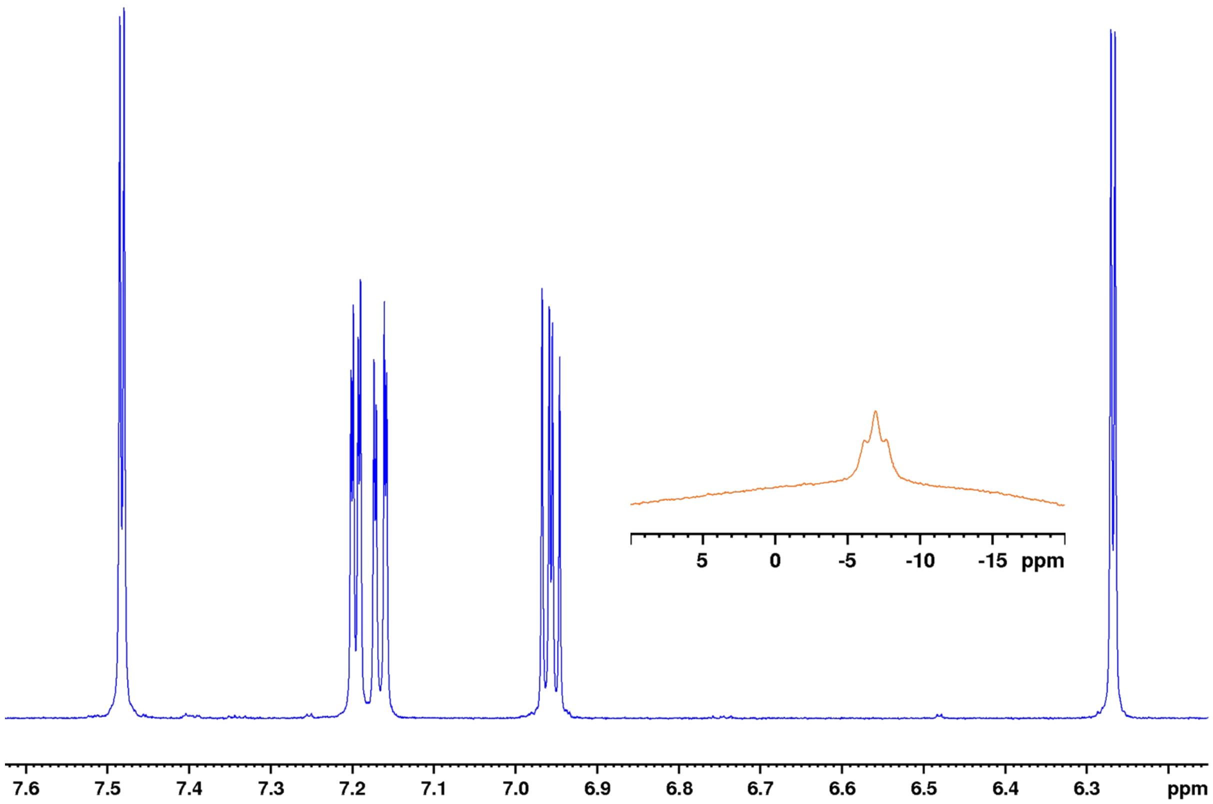

Figure S12. ^{1}H spectrum of **5** (blue), with ^{11}B spectrum insert displaying only triplet peak from **5** (orange).

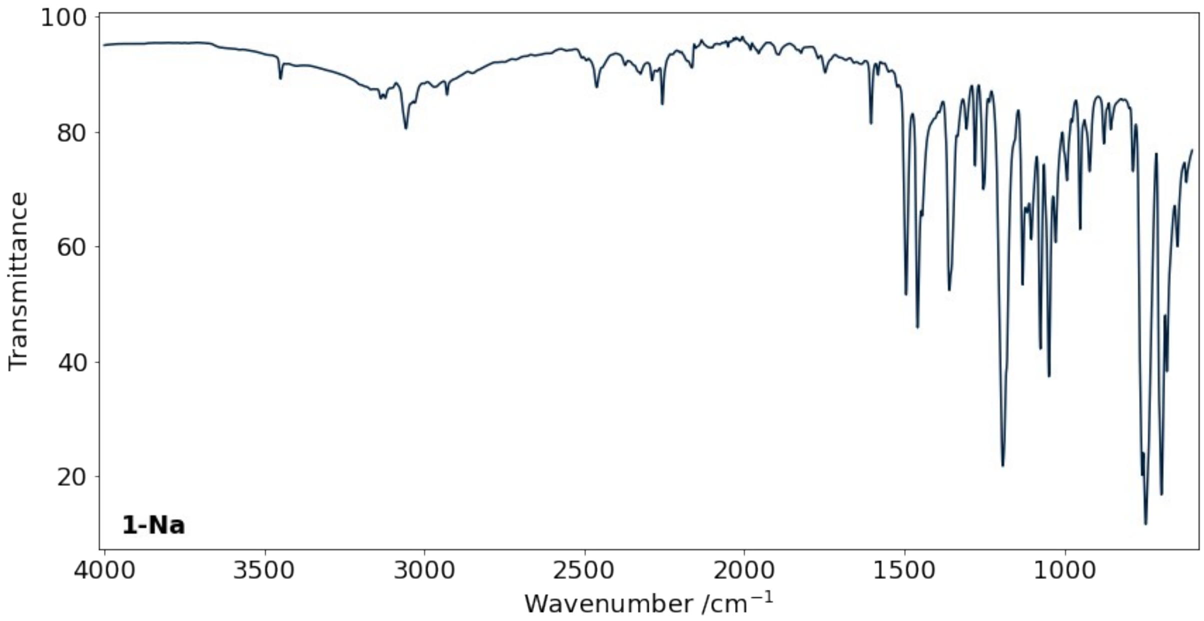

Figure S13. ATR FTIR spectrum of **1-Na**.

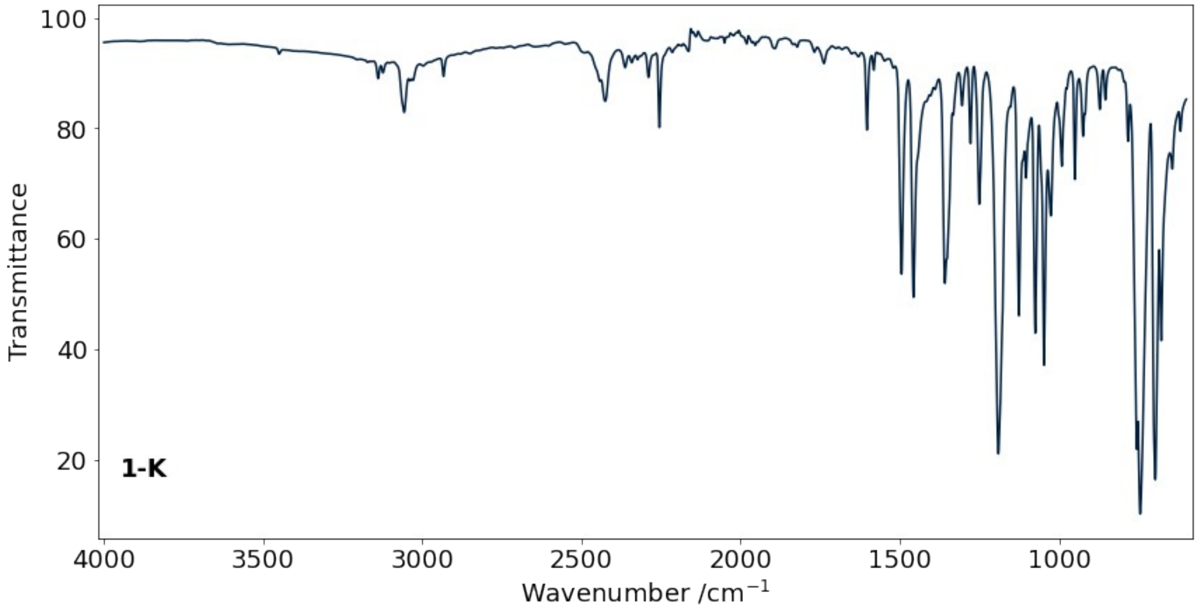

Figure S14. ATR FTIR spectrum of **1-K**.

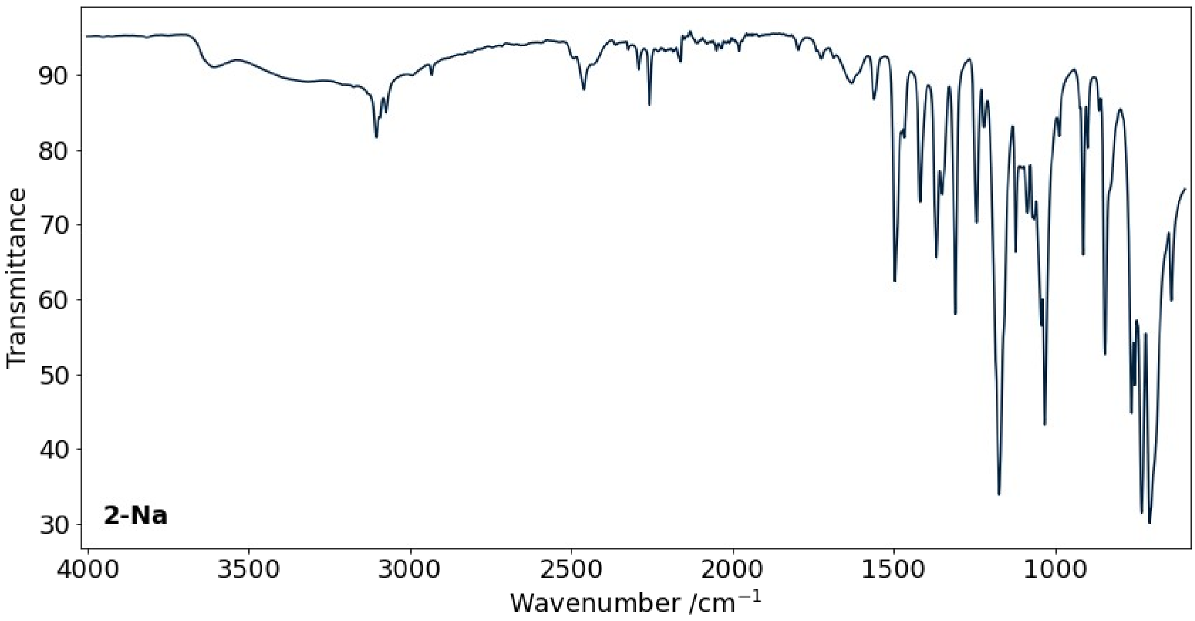

Figure S15. ATR FTIR spectrum of **2-Na.**

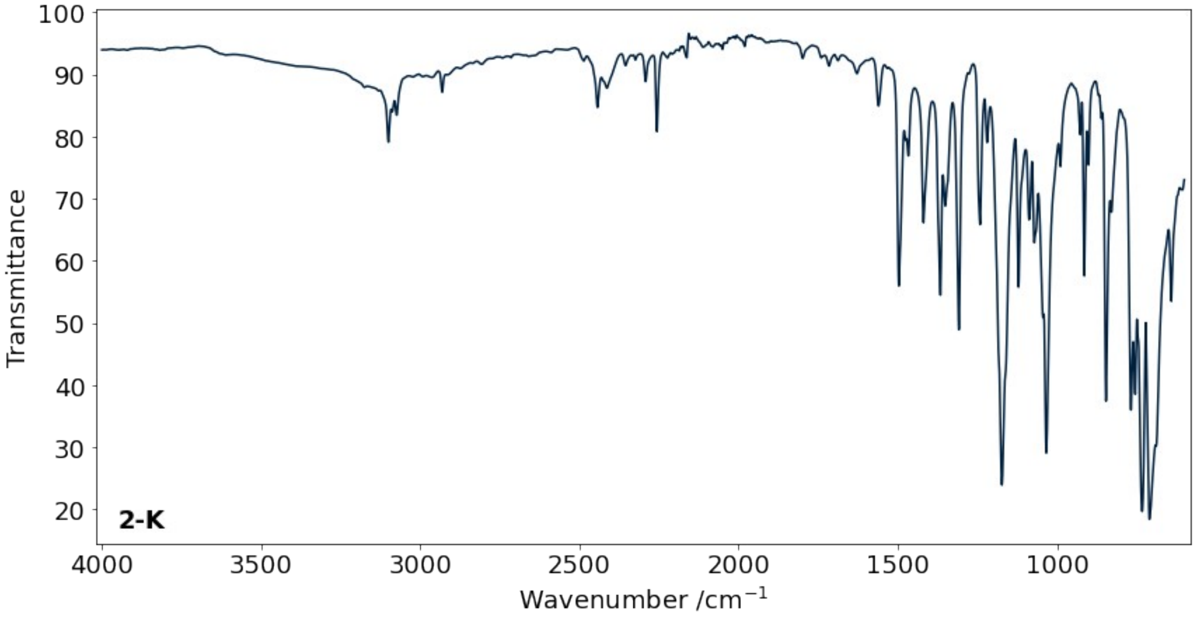

Figure S16. ATR FTIR spectrum of **2-K**.

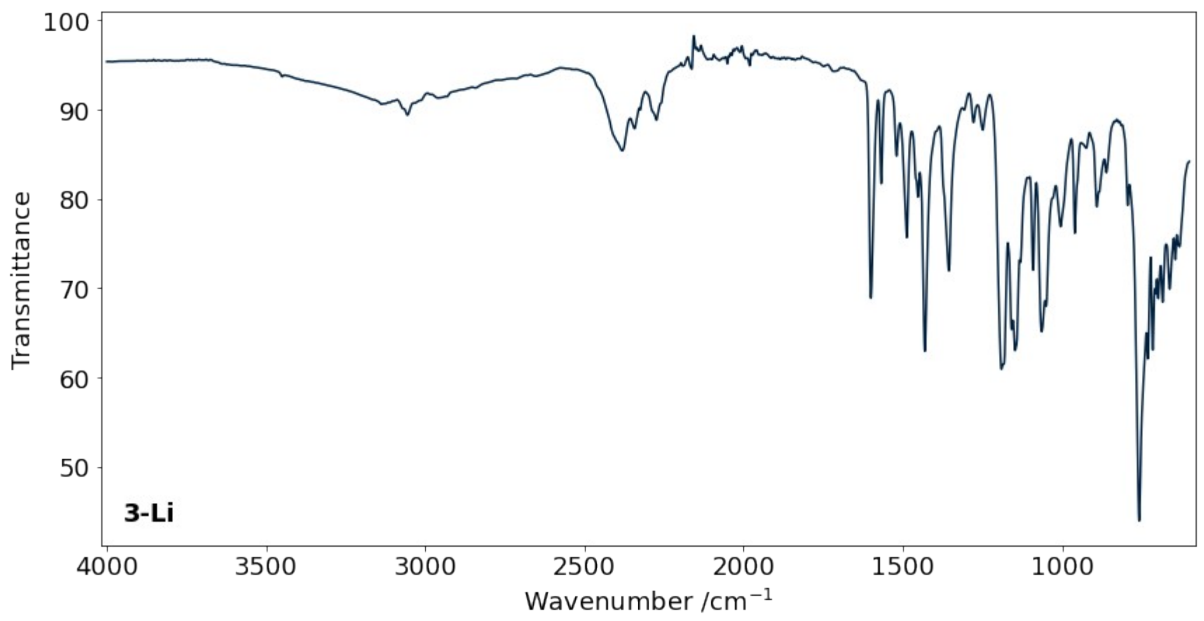

Figure S17. ATR FTIR spectrum of **3-Li**.

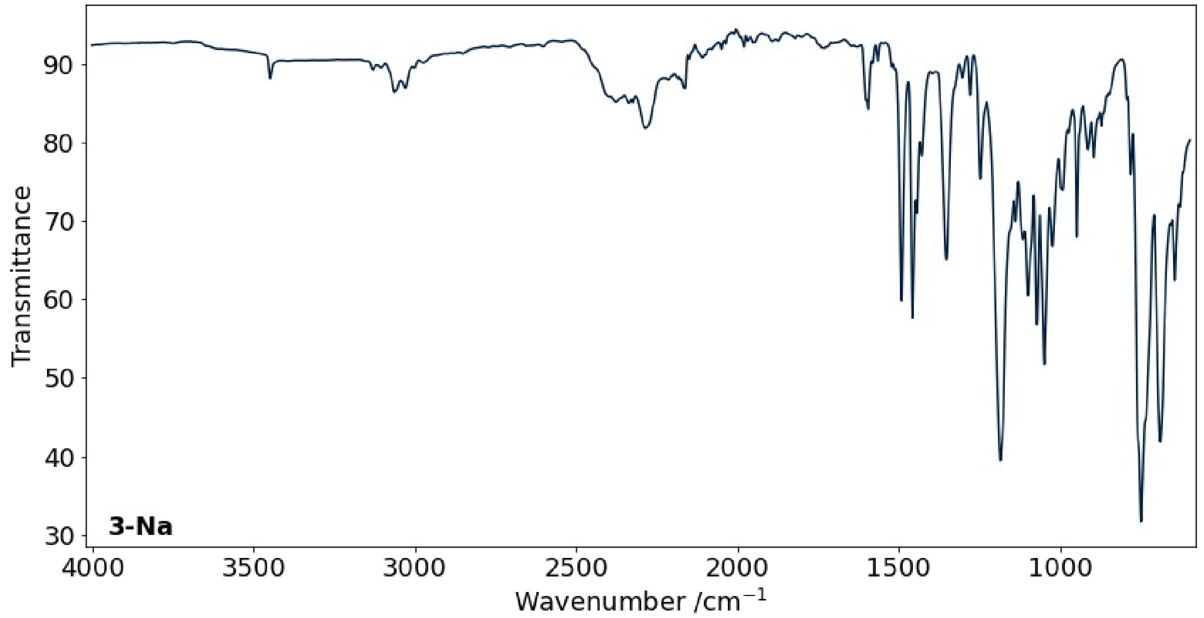

Figure S18. ATR FTIR spectrum of **3-Na**.

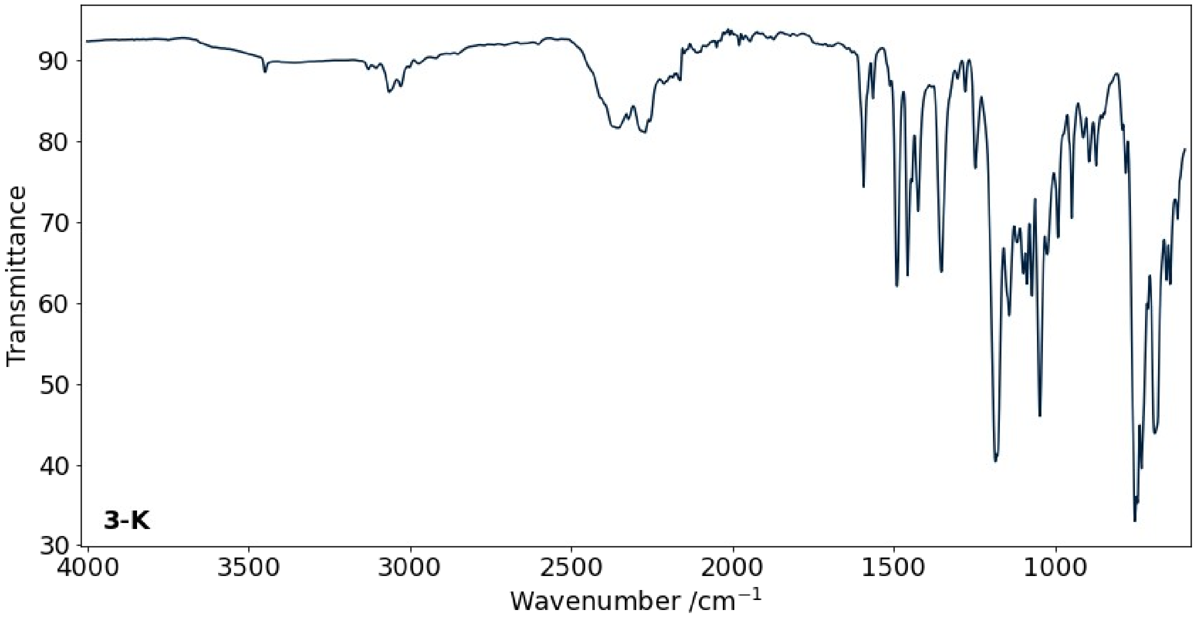

Figure S19. ATR FTIR spectrum of **3-K**.

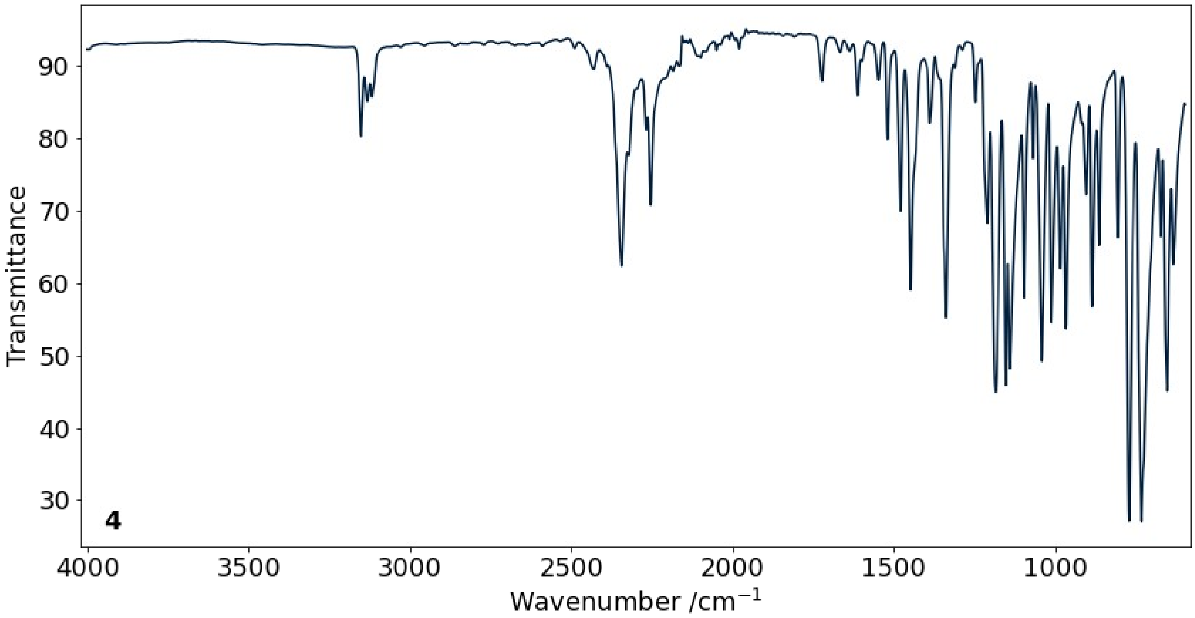

Figure S20. ATR FTIR spectrum of **4**.

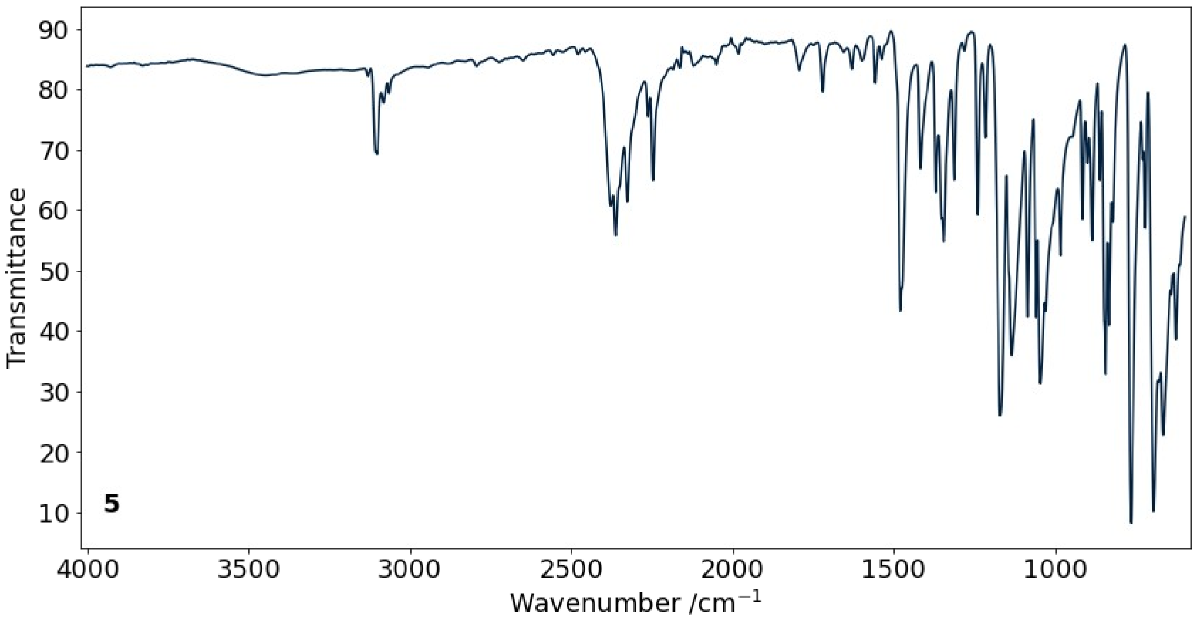

Figure S21. ATR FTIR spectrum of **5**.

3. Single Crystal X-ray Diffraction Collection, Refinement Details and Data

Single crystal X-ray diffraction data for **1-Na**, **2-Na**, **2-K**, **3-Li$_2$**, **3-Na$_2$** and **4** were collected on a Brucker D8 QUEST diffractometer at 100 K, fitted with a CCD area detector employing a mirror-monochromatic MoK_α radiation source (λ = 0.71073 Å). Integration and scaling data of collections were analysed using either or APEX4 software. All data was collected with either exposure times of 10 seconds with 1° frame widths (**1-Na**, **2-Na**, **2-K**, **3-Li$_2$**, **3-Na$_2$**) or 4 seconds with 0.8° frame widths (**4**) on ω and ϕ scans. Multi-scan absorptions corrections were used for all compounds. Data was solved in APEX4 software by intrinsic phasing methods using SHELXL.[2] Refinement of crystal data was carried out using least-square refinement methods employed by SHELXT,[3] with all non-hydrogen atoms having anisotropic displacement parameters. Hydrogen atoms were fixed using the riding model. Olex2-1.3[4] was employed for refining data and molecular graphics using postscript image exports. The following disorder was observed; **1-Na** shows two sites that the MeCN ligand occupies, akin to previous reporting of **1-K**, and **3-Li$_2$** presents disorder in the pyrazolyl substituents and has been split with 0.5 occupancies. All supplementary crystal data sets can be found on the Cambridge Crystal Data Centre under the codes CCDC 2357156-2357161.

Table S1. Crystallographic data for compounds **1-Na, 2-Na**, **2-K**, **3-Li**, **3-Na**, **4**.

	**1-Na**	**2-Na**	**2-K**	**3-Li**	**3-Na**	**4**
Systematic Name	$[Na(Tp^{Ph})(MeCN)_3]$	$[Na(Tp^{2\text{-}Th})(MeCN)_3]$	$[K(Tp^{2\text{-}Th})(MeCN)_3]$	$[Li(\mu\text{-}Bp^{2\text{-}py})]_2$	$[Na(\mu\text{-}Bp^{2\text{-}py})]_2$	$[K(Bp^{2\text{-}Fu})]_n$
Formula	$C_{33}H_{31}BN_9Na$	$C_{27}H_{25}BN_9NaS_3$	$C_{27}H_{25}BKN_9S_3$	$C_{32}H_{16}B_2N_{12}Li_2$	$C_{32}H_{16}B_2N_{12}Na_2$	$[C_{14}H_{12}BKN_4O_2]_n$
MW/ g mol^{-1}	587.47	605.54	621.65	616.16	648.26	318.19
Crystal syst	trigonal	trigonal	trigonal	monoclinic	monoclinic	orthorhombic
Space Group	$P\bar{3}$	*R*3*c*	*R*3*c*	*C*2/*c*	*C*2/*c*	*Pnma*
a /Å	14.9362(3)	17.1463(5)	17.4388(8)	10.5827(5)	15.7237(10)	11.7918(6)
b /Å	14.9362(3)	17.1463(5)	17.4388(8)	21.2856(11)	10.8738(7)	23.2058(13)
c /Å	8.0716(3)	17.0555(7)	16.8446(7)	14.6160(6)	20.0182(11)	5.2271(3)
α /°	90	90	90	90	90	90
β /°	90	90	90	104.567(2)	111.251(2)	90
γ /°	120	120	120	90	90	90
V /Å^3	1559.45(9)	4342.5(3)	4436.3(4)	3186.6(3)	3189.9(3)	1430.33(14)
Z	2	6	6	4	4	4
ρ /g cm^{-3}	1.251	1.389	1.396	1.284	1.350	1.478
F(000)	616	1884	1932	1280	1344	656
μ /mm^{-1}	0.090	0.307	0.427	0.080	0.109	0.383
Reflections Collected	19605	23363	98652	72157	35586	39510
R_{int} (R_σ)	0.0520(0.0272)	0.0440(0.0249)	0.0540(0.0174)	0.0810(0.0253)	0.0549(0.0284)	0.0540(0.0190)
Data/Restraints/Parameters	2580/0/144	2967/1/125	2390/1/126	3249/0/279	3661/0/217	1797/0/109
R indexes ($I \geq 2\sigma(I)$)	R_1 = 0.0439<br>wR_2 = 0.0983	R_1 = 0.0343<br>wR_2 = 0.0782	R_1 = 0.0452<br>wR_2 = 0.0962	R_1 = 0.0610<br>wR_2 = 0.1429	R_1 = 0.0571<br>wR_2 = 0.1335	R_1 = 0.0313<br>wR_2 = 0.0747
R indexes (all data)	R_1 = 0.0603<br>wR_2 = 0.1156	R_1 = 0.0399<br>wR_2 = 0.0852	R_1 = 0.0605<br>wR_2 = 0.1122	R_1 = 0.0870<br>wR_2 = 0.1817	R_1 = 0.0767<br>wR_2 = 0.1616	R_1 = 0.0349<br>wR_2 = 0.0786
Diff. peak/hole /*e* Å^{-3}	0.30/-0.30	0.28/-0.26	0.34/-0.55	0.22/-0.33	0.40/-0.38	0.33/-0.26
Goodness-of-fit on F^2	1.099	1.180	1.267	1.157	1.126	1.109
Flack Parameter		0.04(2)	-0.022(14)			

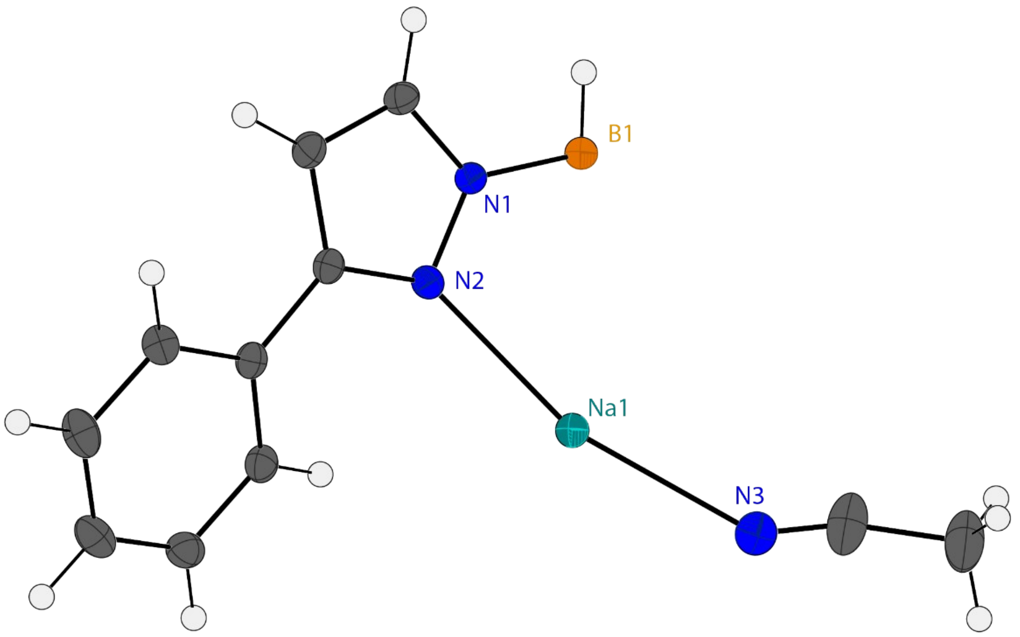

Figure S22. Asymmetric unit of **1-Na**, with selected atoms labelled (carbon = grey, hydrogen = white), shown at

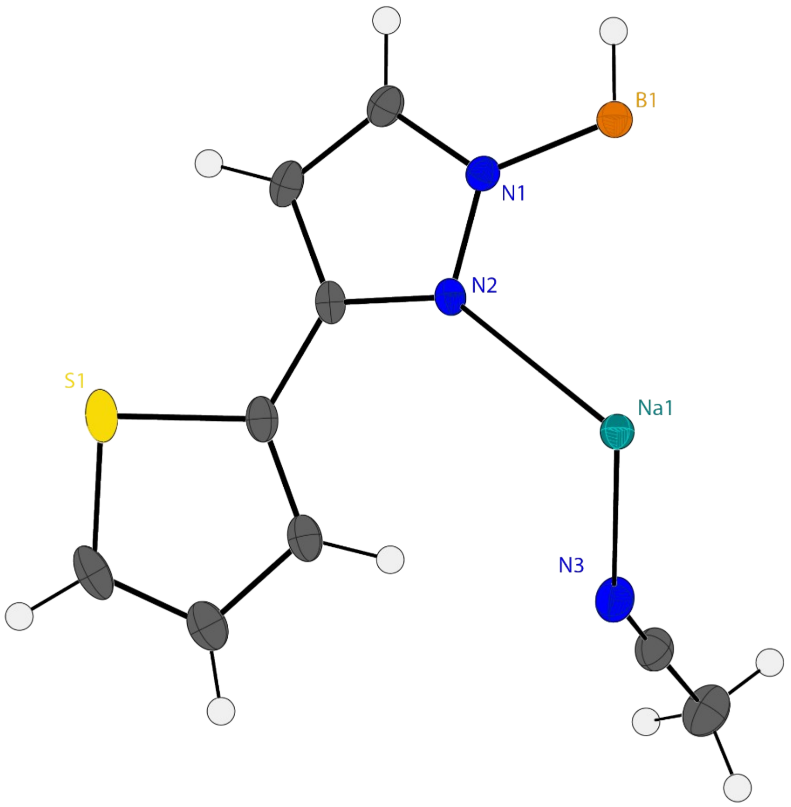

50% thermal ellipsoids.

Figure S23. Asymmetric unit of **2-Na**, with selected atoms labelled (carbon = grey, hydrogen = white), shown at 50% thermal ellipsoids.

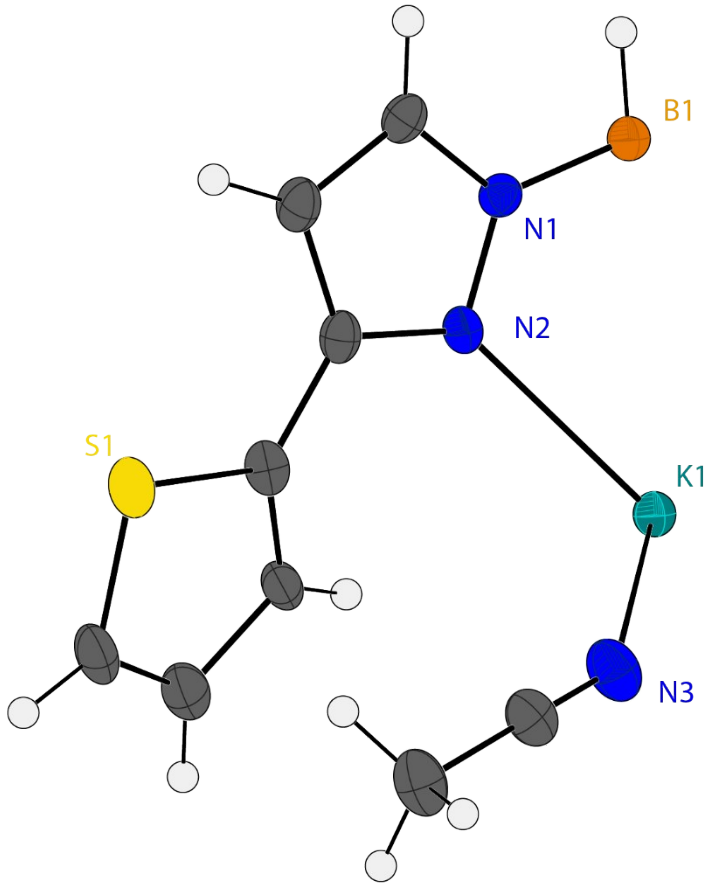

Figure S24. Asymmetric unit of **2-K**, with selected atoms labelled (carbon = grey, hydrogen = white), shown at 50% thermal ellipsoids.

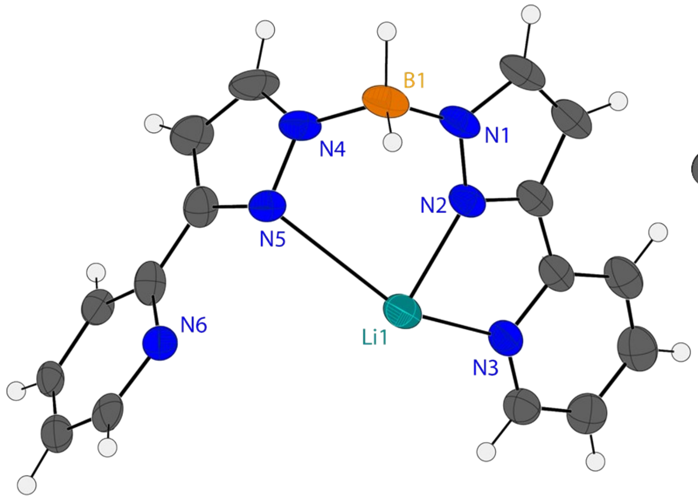

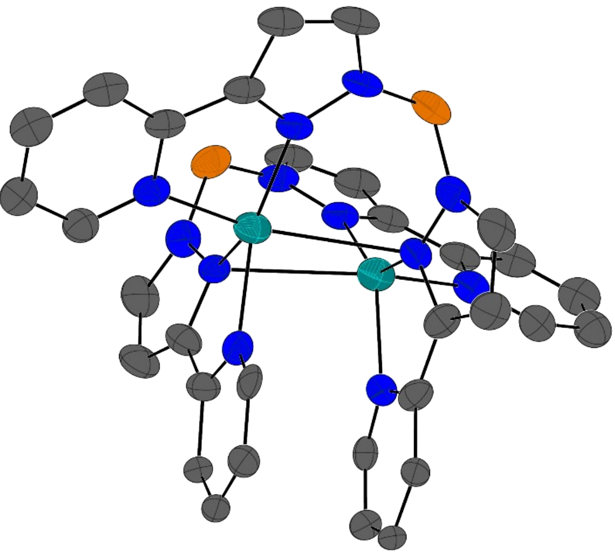

Figure S25. Asymmetric unit (left) and dimeric structure (right) of **3-Li$_2$**, with selected atoms labelled (carbon = grey, hydrogen = white), shown at 50% thermal ellipsoids.

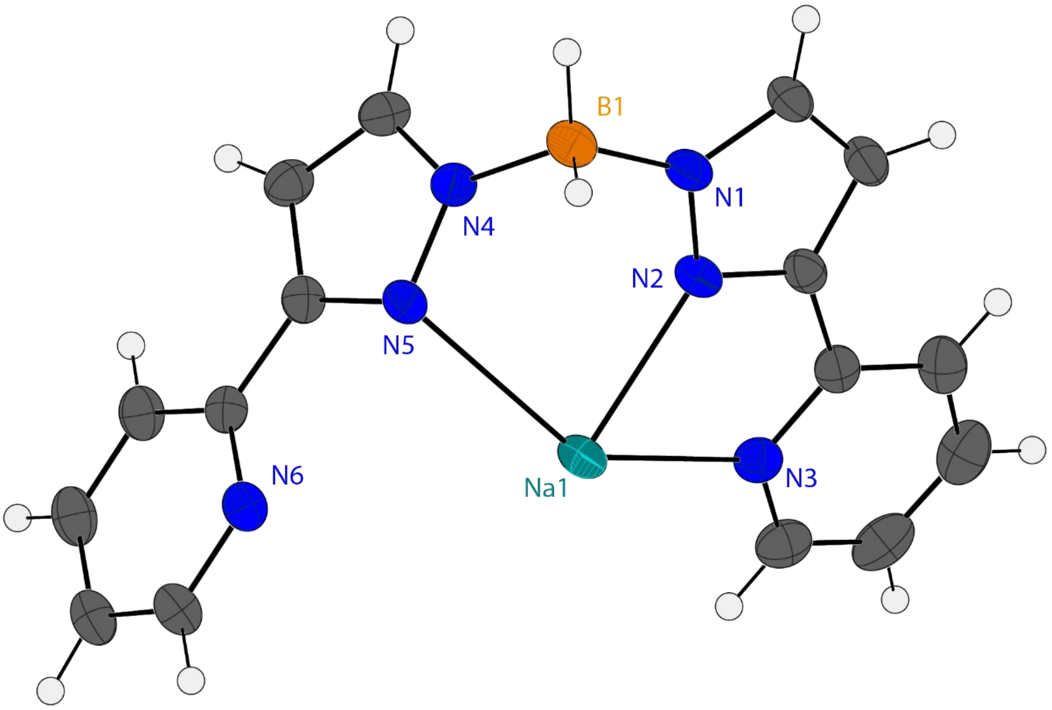

Figure S26. Asymmetric unit of **3-Na$_2$**, with selected atoms labelled (carbon = grey, hydrogen = white), shown

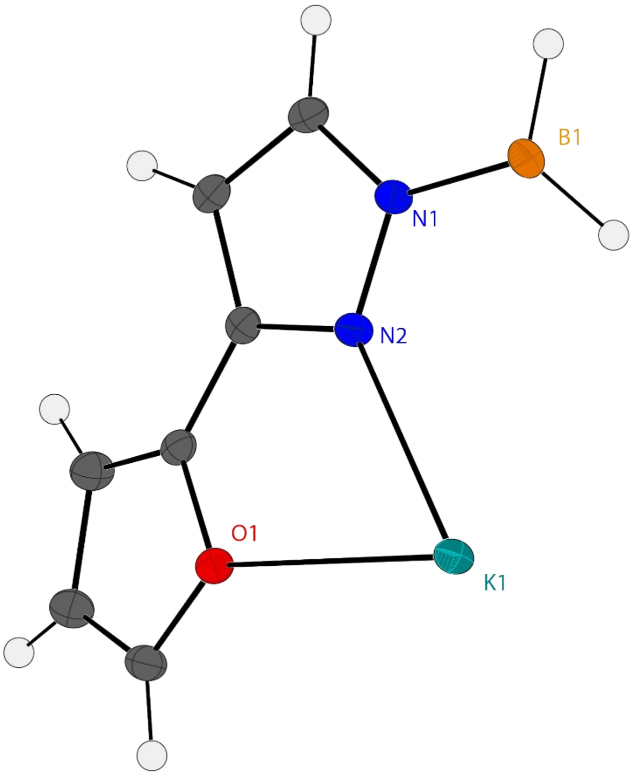

at 50% thermal ellipsoids.

Figure S27. Asymmetric unit of **4**, with selected atoms labelled (carbon = grey, hydrogen = white), shown at 50% thermal ellipsoids.

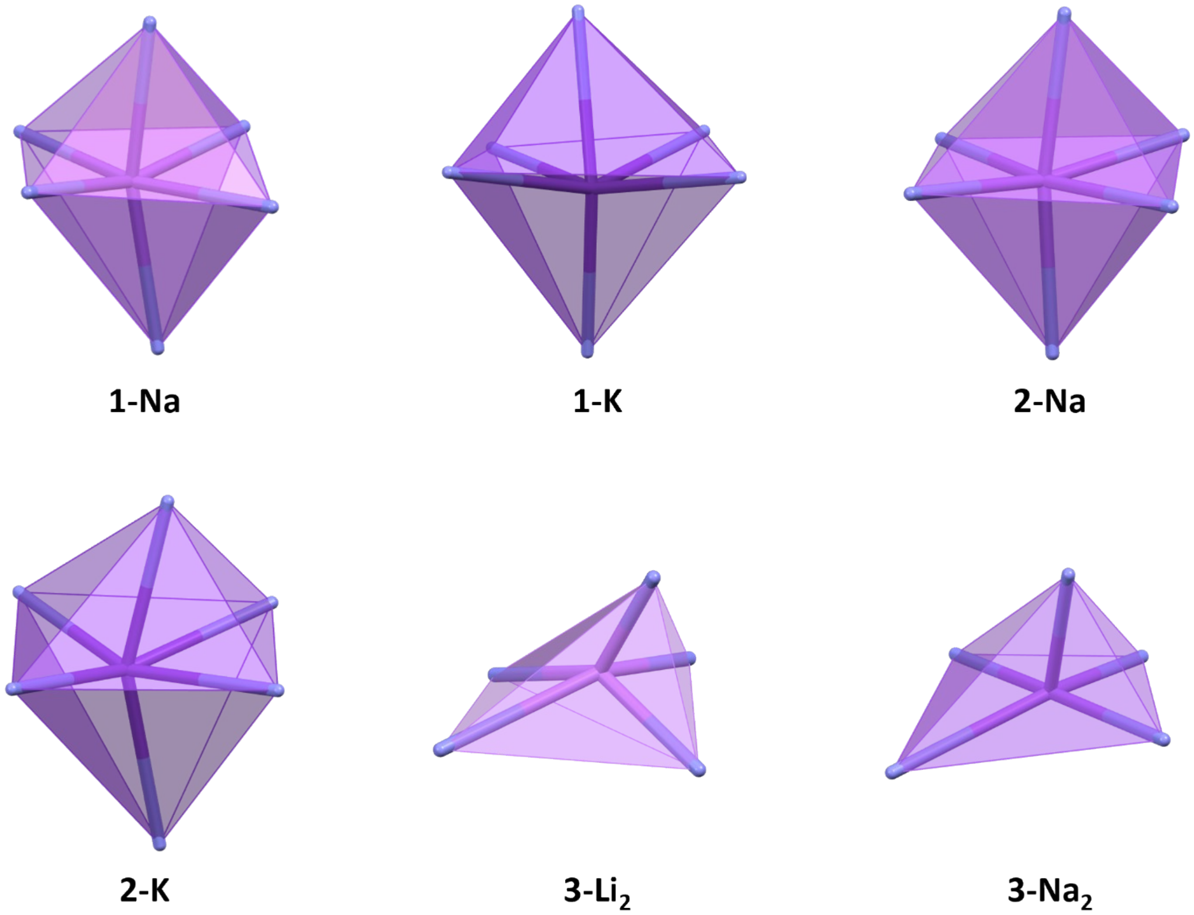

Figure S28. Coordination environments showcasing pseudo-octahedral (**1-Na**, **1-K**, **2-Na**, **2-K**) and pseudo square pyramidal (**3-Li$_2$**, **3-Na$_2$**)

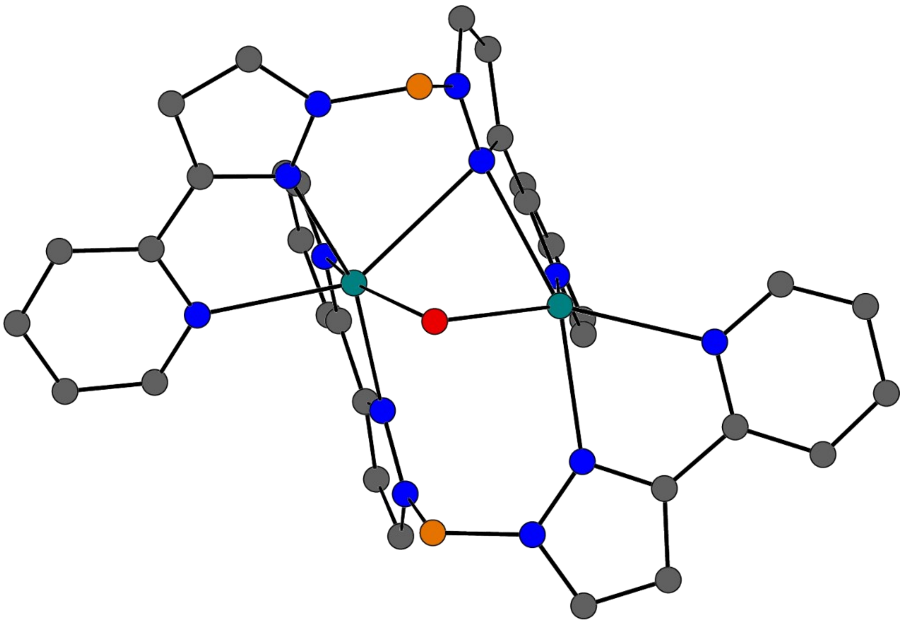

Figure S29. Dimeric solid-state structure of [{Na(μ-Bp$^{2\text{-py}}$)}$_2$)(μ-OH$_2$)] show casing the hydration that occurs when recrystallising from solvents that are not dried. Sodium = teal, oxygen = red, nitrogen = blue, carbon = grey, boron = orange, hydrogen atoms and lattice solvent molecules of CH_2Cl_2 have been omitted for clarity.

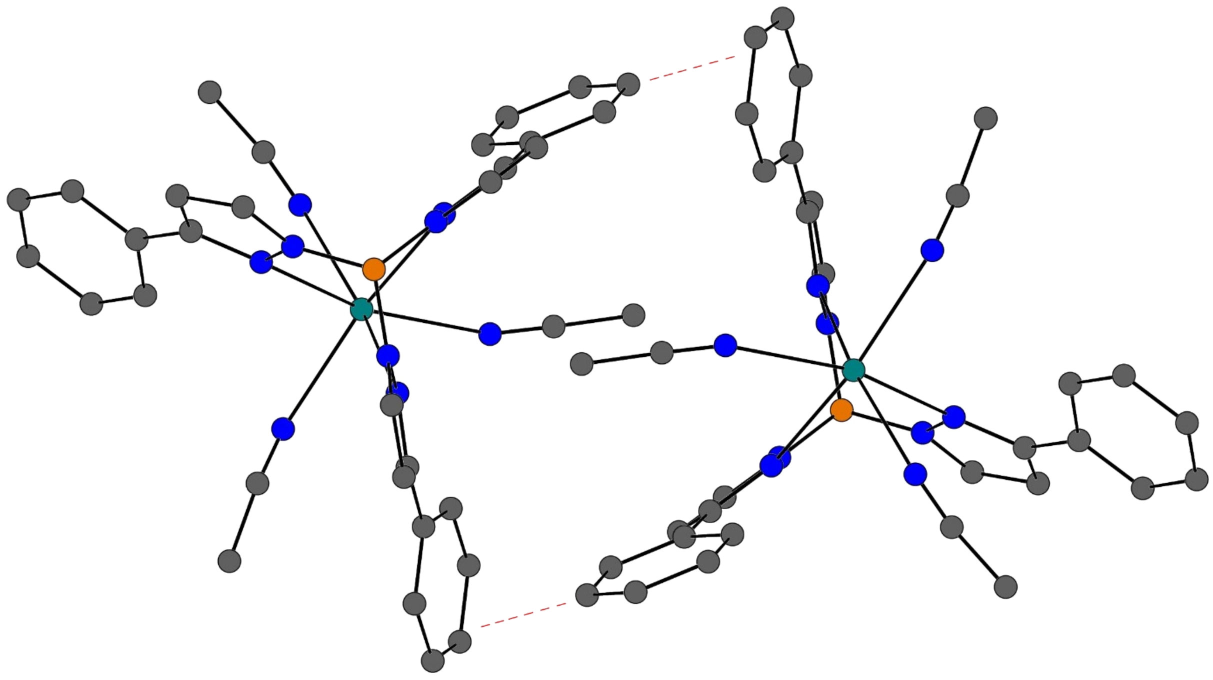

Figure S30. Solid-state packing of **1-Na** showcasing the edge-to-face π-stacking (dashed red lines) between the columns along the *c*-axis.

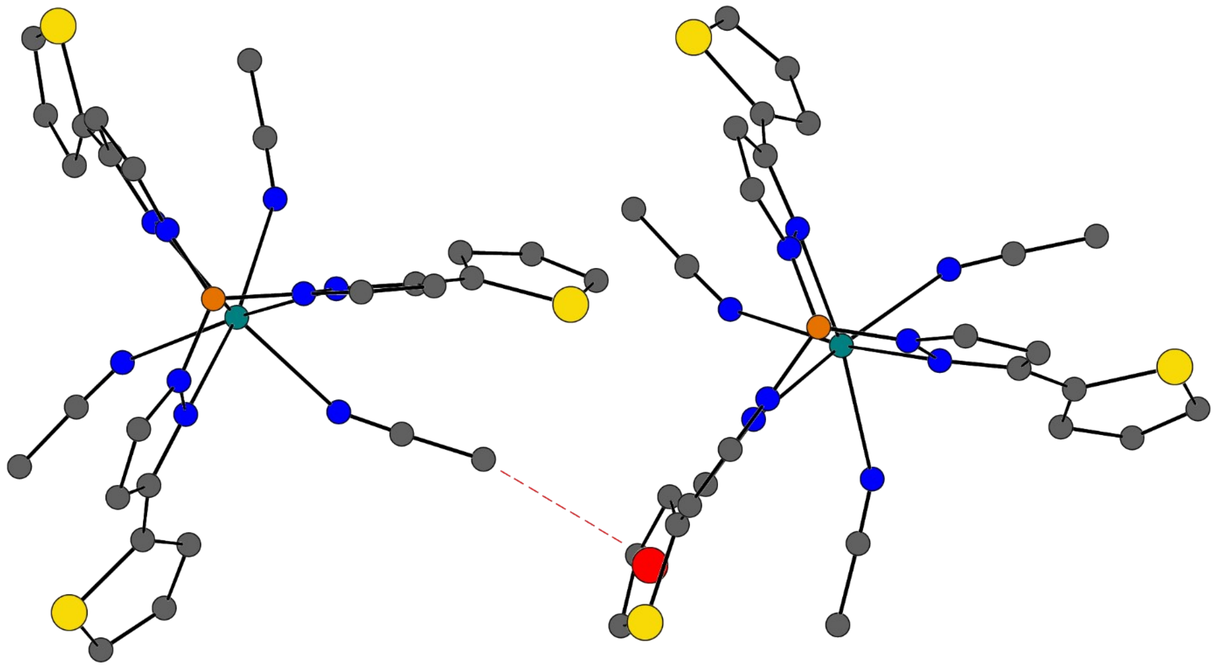

Figure S31. Solid-state packing of **2-Na** showcasing the edge-on p-interaction (dashed red line) between adjacent molecules between the centroid of the thienyl group (red sphere) and the terminal carbon of MeCN.

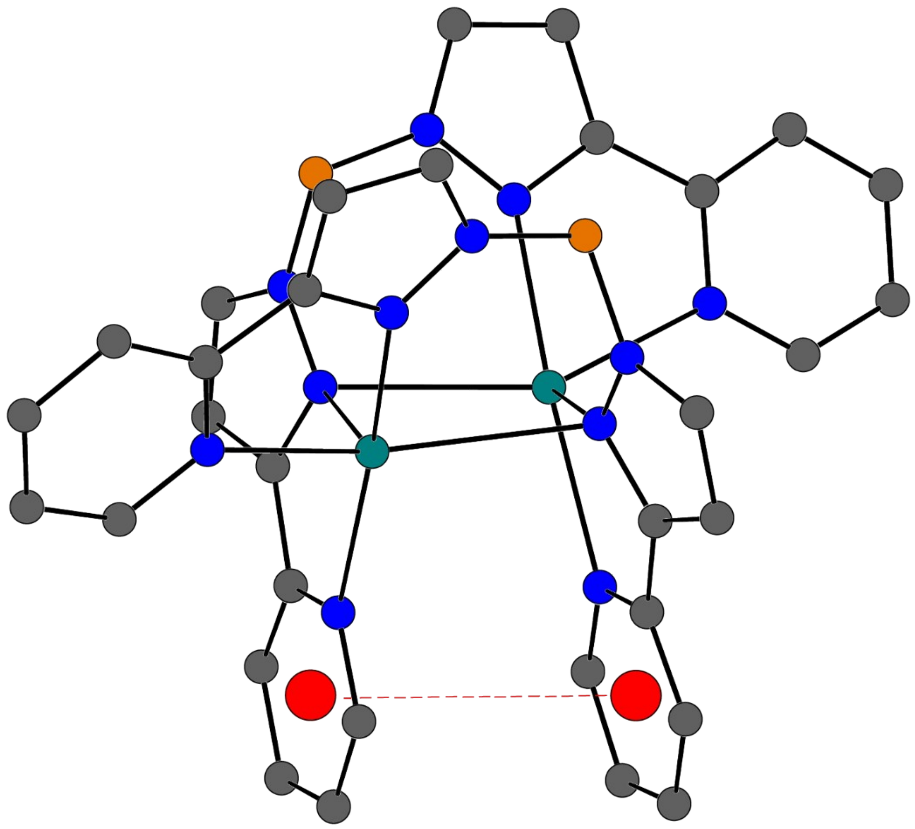

Figure S32. Intramolecular π-π stacking (dashed red line) between pyridyl centroids (red spheres) seen in **3-Na$_2$**.

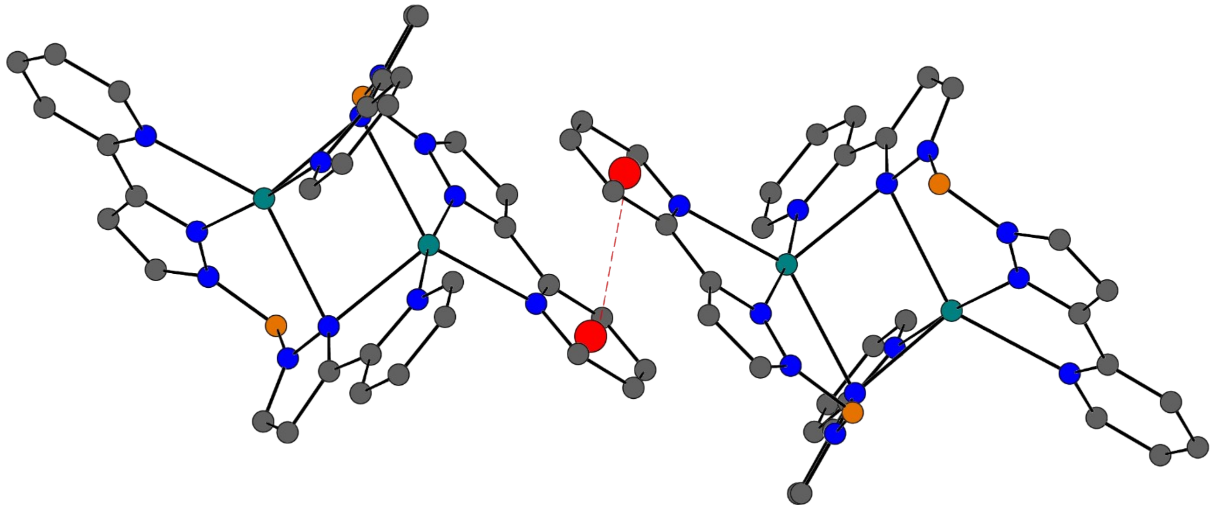

Figure S33. Intermolecular π-π stacking (dashed red line) between pyridyl centroids (red spheres) seen in the solid-state packing of **3-Na$_2$**.

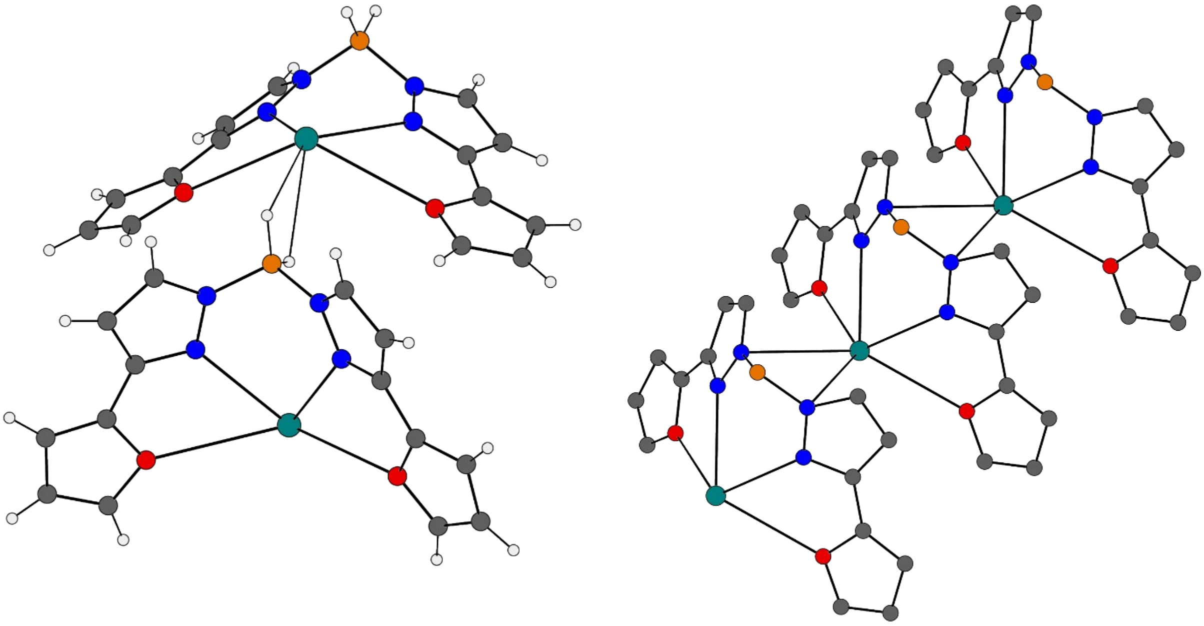

Figure S34. Solid-state packing of **4**, revealing the two-dimensional coordination polymer structure extending in the *a*-direction *via* the borohydrides (left) and the *c*-direction through pyrazolyl substituents (right).

4. SHAPE2.1 Results

Table S3. CShM values for **3-Li₂** and **3-Na₂** when M1-N5[1] bond is included (5 coordinate and metal centre).

	PP-5	vOC-5	TBPY-5	SPY-5	JTBPY-5
**3-Li_2**	24.177	5.931	7.113	4.829	6.607
**3-Na_2**	21.803	5.668	7.238	5.785	10.356

Table S4. CShM values for **3-Li_2** and **3-Na_2** when M1-N5[1] bond is not included (4 coordinate and metal centre).

	SP-4	T-4	SS-4	vTBPY-4
**3-Li_2**	22.786	7.932	6.335	6.138
**3-Na_2**	15.136	15.267	5.368	14.464

5. References

1. Amoroso A. J.; Cargill Thompson A. M.; Jeffery J. C.; Jones, P. L.; McCleverty J. A.; Ward M. D., *J. Chem. Soc., Chem. Commun.*, **1994**, 2751-2757.
2. Sheldrick, G. M., *Acta Cryst.*, **2015**, A71, 3-8.
3. Sheldrick, G. M., *Acta Cryst.*, **2008**, A64, 112-122.
4. Dolomanov, O.V., Bourhis, L.J., Gildea, R.J, Howard, J.A.K. & Puschmann, H., *J. Appl. Cryst.*, **2009**, 42, 339-341.
